# Supplementary material for: Inter-nesting area use, migratory routes, and foraging grounds for hawksbill turtles (Eretmochelys imbricata) in the Western Caribbean
Source: PLoS One. 2025 Mar 12;20(3):e0317778. doi: 10.1371/journal.pone.0317778 (PMC11902275; doi:10.1371/journal.pone.0317778)
Supplement: Attachment — Submitted filename: Responses to Reviewers.pdf [file pone.0317778.s003.pdf]

PONE-D-24-40236
Inter-Nesting Area Use, Migratory Routes, and Foraging Grounds for Hawksbill Turtles (Eretmochelys imbricata) in the Western Caribbean
PLOS ONE

Dear Dr. Dunbar,

Thank you for submitting your manuscript to PLOS ONE. After careful consideration, we feel that it has merit but does not fully meet PLOS ONE's publication criteria as it currently stands. Therefore, we invite you to submit a revised version of the manuscript that addresses the points raised during the review process.

Thank you for your patience during the review process. We have now received feedback from two reviewers. One reviewer is well-versed in Hawksbill turtles, while the other has extensive knowledge of satellite tracking in sea turtles. Both reviewers responded positively to the manuscript, highlighting that the study provides valuable insights into the species' biology. I also found the study straightforward, with data clearly presented.

We thank you, Dr. Fujiwara, for your oversight of the review process for our manuscript. We understand that sometimes the process can take additional time due to the busy schedule of Reviewers (of which I myself am one for several journals). We appreciate the positive feedback provided by both the Reviewers, as well as yourself.

However, some values in the manuscript lack associated error terms, and certain error terms require clarification regarding their type (e.g., standard error, standard deviation, confidence interval, or range). Please ensure that error terms are included for all average values and specify their nature. This applies particularly to values presented in the tables. Additionally, the tables appear to be cut off on the right side; please verify that they are formatted correctly. There may also be some issues with the figures, as noted by one of the reviewers.

We have carefully reviewed all sections in which we have omitted error terms, as well as reported errors, but had not clearly distinguished standard error from standard deviation, confidence intervals, or ranges. We have provided clarification for each of these, as well as included error terms where they had mistakenly previously been omitted. We also included a statement at the end of the methods section on L258 to clarify that all means are reported with ± 1SD. We appreciate this comment, as it will provide clarification for readers of the paper after publication.

Finally, please do not forget to review comments made directly to the manuscript (see attached document).

The comments provided by the Reviewer in the attached manuscript as Track Changes, have been addressed, and are also reported in this Responses to Reviewers document on a line by line basis.

L98 – 99 the Reviewer suggested some slight changes in text. These were style changes, and we have gone ahead and edited the style changes as suggested.

Lines 142 and 161, Reviewer asked for the lengths of the specific beaches on which the satellite attachments took place. We added "…1 km long…" to L142, and "This includes some 10 km of beach which annually supports sea turtle nesting." to L161.

On L187 a Reviewer asked for clarification on which shoulder PIT tags were applied to nesting turtles. The standard procedure for the injection of PIT tags into nesting turtles is in the right or left shoulder. Since different projects across this study maintain different protocols, we elected to have the sentence remain as it was originally written. The placement of the PIT tag is inconsequential to the focus of the paper; that being satellite tracking.

We thank the Reviewer for catching that Kevlar should have a capital "K" on L198. We have corrected this error.

L208 (Table 1). The Reviewer pointed out that the Tables (1 and 2) run past the margin on the right side of the page and therefore are not viewable. We attempted to place page breaks before and after each Table (placing them on their own pages) and turned these pages to Landscape orientation. However, this attempt caused further issues to the readability of the Tables. Instead, we have additionally uploaded each Table as a separate document in the resubmission process, hoping this will allow the editorial team of the journal to edit and place the tables where they deem appropriate for the publication.

L258. The Reviewer asked for us to clarify if we were using Standard Error or Standard Deviation for the reporting of means throughout the paper. We have clarified this by making a general statement at the end of the Methods section (L258) that states for all results we report means ± 1 SD.

Although not specified by the Reviewer, we changed the text slightly in L390 to pluralize UD to UDs and changed the word "and" to "to" for the range of UDs in that sentence.

The Reviewer suggested that the statement from L427 – 431 (Fig. 9 legend) that is repeated in Figs. 10, and 11, be consolidated and placed at the start of the section entitled "Influence of currents on turtle movements." We have removed this repeated statement from each of the figures and now placed it in L408 – 411, and thank the Reviewer for this helpful suggestion.

On L482, the Reviewer recommended a very slight change of text, from "With this report…" to "Here…" We have made that slight change to the wording, although this is simply a choice of style.

In L693 – 698, we revised the acknowledgements slightly to include additional permit and IACUC numbers for the different projects, as appropriate, and have added in an acknowledgment to the two Reviewers. We have left the MRG and ProTECTOR, Inc. Contribution Numbers blank until we receive confirmation of acceptance of the manuscript for publication in PLOS ONE, and will add these numbers in at the Proof stage.

Please submit your revised manuscript by Dec 26 2024 11:59PM. If you will need more time than this to complete your revisions, please reply to this message or contact the journal office at plosone@plos.org. When you're ready to submit your revision, log on to https://www.editorialmanager.com/pone/ and select the 'Submissions Needing Revision' folder to locate your manuscript file.

Please include the following items when submitting your revised manuscript:

- A rebuttal letter that responds to each point raised by the academic editor and reviewer(s). You should upload this letter as a separate file labeled 'Response to Reviewers'.
- A marked-up copy of your manuscript that highlights changes made to the original version. You should upload this as a separate file labeled 'Revised Manuscript with Track Changes'.
- An unmarked version of your revised paper without tracked changes. You should upload this as a separate file labeled 'Manuscript'.

If you would like to make changes to your financial disclosure, please include your updated statement in your cover letter. Guidelines for resubmitting your figure files are available below the reviewer comments at the end of this letter.

If applicable, we recommend that you deposit your laboratory protocols in protocols.io to enhance the reproducibility of your results. Protocols.io assigns your protocol its own identifier (DOI) so that it can be cited independently in the future. For instructions see: https://journals.plos.org/plosone/s/submission-guidelines#loc-laboratory-protocols. Additionally, PLOS ONE offers an option for publishing peer-reviewed Lab Protocol articles, which describe protocols hosted on protocols.io. Read more information on sharing protocols at https://plos.org/protocols?utm_medium=editorial-email&utm_source=authorletters&utm_campaign=protocols.

We look forward to receiving your revised manuscript.

Kind regards,

Masami Fujiwara, PhD
Academic Editor
PLOS ONE

Journal requirements:

When submitting your revision, we need you to address these additional requirements.

1. Please ensure that your manuscript meets PLOS ONE's style requirements, including those for file naming. The PLOS ONE style templates can be found at https://journals.plos.org/plosone/s/file?id=wjVg/PLOSOne_formatting_sample_main_body.pdf and https://journals.plos.org/plosone/s/file?id=ba62/PLOSOne_formatting_sample_title_authors_affiliations.pdf

2. We note that you have indicated that there are restrictions to data sharing for this study. PLOS only allows data to be available upon request if there are legal or ethical restrictions on sharing data publicly. For more information on unacceptable data access restrictions, please see http://journals.plos.org/plosone/s/data-availability#loc-unacceptable-data-access-restrictions.

Before we proceed with your manuscript, please address the following prompts:

a) If there are ethical or legal restrictions on sharing a de-identified data set, please explain them in detail (e.g., data contain potentially identifying or sensitive patient information, data are owned by a third-party organization, etc.) and who has imposed them (e.g., a Research Ethics Committee or Institutional Review Board, etc.). Please

also provide contact information for a data access committee, ethics committee, or other institutional body to which data requests may be sent.

b) If there are no restrictions, please upload the minimal anonymized data set necessary to replicate your study findings to a stable, public repository and provide us with the relevant URLs, DOIs, or accession numbers. For a list of recommended repositories, please see https://journals.plos.org/plosone/s/recommended-repositories. You also have the option of uploading the data as Supporting Information files, but we would recommend depositing data directly to a data repository if possible.

We will update your Data Availability statement on your behalf to reflect the information you provide.

3. Please review your reference list to ensure that it is complete and correct. If you have cited papers that have been retracted, please include the rationale for doing so in the manuscript text, or remove these references and replace them with relevant current references. Any changes to the reference list should be mentioned in the rebuttal letter that accompanies your revised manuscript. If you need to cite a retracted article, indicate the article's retracted status in the References list and also include a citation and full reference for the retraction notice.

[Note: HTML markup is below. Please do not edit.]

Reviewers' comments:

Reviewer's Responses to Questions

Comments to the Author

1. Is the manuscript technically sound, and do the data support the conclusions?

The manuscript must describe a technically sound piece of scientific research with data that supports the conclusions. Experiments must have been conducted rigorously, with appropriate controls, replication, and sample sizes. The conclusions must be drawn appropriately based on the data presented.

Reviewer #1: Yes

Reviewer #2: Yes

---

2. Has the statistical analysis been performed appropriately and rigorously?

Reviewer #1: Yes

Reviewer #2: Yes

---

3. Have the authors made all data underlying the findings in their manuscript fully available?

The PLOS Data policy requires authors to make all data underlying the findings described in their manuscript fully available without restriction, with rare exception (please refer to the Data Availability Statement in the manuscript PDF file). The data should be provided as part of the manuscript or its supporting information, or deposited to a public repository. For example, in addition to summary statistics, the data points behind means, medians and variance measures should be available. If there are restrictions on publicly sharing data—e.g. participant privacy or use of data from a third party—those must be specified.

Reviewer #1: Yes

Reviewer #2: Yes

---

4. Is the manuscript presented in an intelligible fashion and written in standard English?

PLOS ONE does not copyedit accepted manuscripts, so the language in submitted articles must be clear, correct, and unambiguous. Any typographical or grammatical errors should be corrected at revision, so please note any specific errors here.

Reviewer #1: Yes

Reviewer #2: Yes

---

5. Review Comments to the Author

Please use the space provided to explain your answers to the questions above. You may also include additional comments for the author, including concerns about dual publication, research ethics, or publication ethics. (Please upload your review as an attachment if it exceeds 20,000 characters)

Reviewer #1: This paper provides valuable insight into the inter-nesting, and foraging areas, and migratory routes of hawksbill turtles in a region for which there is a definite lack of information. I conducted satellite tagging of loggerhead and greens turtles for a number of years and fully aware of the effort and field conditions involved. I hope the data gathered in this project will assist resource managers in better defining areas that need additional protection from the impacts of commercial fishing activities, both legal and illegal. The paper is well-written and with a few minor editorial changes is good to go.

We thank Reviewer #1 for the very positive and encouraging review comments. We have considered and addressed each editorial change that Reviewers have provided.

Reviewer #2: Overall a well written, clean MS. I have no comments that would substantively change the MS that would not be a matter of personal preference only.
The figs attached to the end of the document are in the wrong order - if this is published make sure they are arranged in the order you want.

We extend our thanks to Reviewer #2 for their positive response to the manuscript, and kind direction in improving the manuscript. We have ensured that the order of the figures has been corrected (this was likely an oversight during the online submission process).

At L131 (Fig. 1 legend) Reviewer #2 suggested that we include the number of satellite tags deployed within the individual insets of the figure. However, we did not see this as necessary since the number of satellite tags deployed (n) at each study site is clearly listed in the figure legend, and each figure inset is clearly labelled with the corresponding country location.

L138 – Reviewer 2 suggested including the length of the study site beaches. These have been added in their respective sections. For Pumpkin Hill Beach, Honduras: L142; for Gandoca-Manzanillo National Wildlife Refuge, Costa Rica: L161.

L186. The Reviewer asked for clarification on the application of PIT tags (i.e. in the right or left shoulder, or if application varied). Because the project was undertaken in different countries, with different tagging protocols, we elected not to make the suggested change. The placing of PIT tags is consistently done in the right or left shoulder (depending on project-specific protocols), and therefore does not require the clarification of which shoulder PIT tags were placed in this manuscript, since these details are inconsequential to the scope of this report, which is on satellite tracking rather than PIT tagging and tracking.

L197. The Reviewer pointed out that Kevlar is a proper noun and thus, requires capitalization. Thank you. This has now been corrected on L199.

In Figures 9, 10, and 11, Reviewer #2 pointed out the repetition of the same phrasing for each of the figures, and suggested removing those phrases from the figure legends, and instead, placing them at the beginning of the section describing the ‘influence of currents on turtle movements’ in order to reduce the redundancy of the phrasing in each figure. We have now done this and placed the description of the colors in L408 – 411, and removed this phrasing from each of Figures 9, 10, and 11.

As suggested, we replaced the phrase “With the current report,” on L475, with the word “Here,” now on L482.

6. PLOS authors have the option to publish the peer review history of their article (what does this mean?). If published, this will include your full peer review and any attached files.

If you choose "no", your identity will remain anonymous but your review may still be made public.

Do you want your identity to be public for this peer review? For information about this choice, including consent withdrawal, please see our Privacy Policy.

Reviewer #1: **Yes:** David S. Addison, Senior Biologist, Conservancy of Southwest Florida, Naples, FL

Reviewer #2: No

[NOTE: If reviewer comments were submitted as an attachment file, they will be attached to this email and accessible via the submission site. Please log into your account, locate the manuscript record, and check for the action link "View Attachments". If this link does not appear, there are no attachment files.]

While revising your submission, please upload your figure files to the Preflight Analysis and Conversion Engine (PACE) digital diagnostic tool, https://pacev2.apexcovantage.com/. PACE helps ensure that figures meet PLOS requirements. To use PACE, you must first register as a user. Registration is free. Then, login and navigate to the UPLOAD tab, where you will find detailed instructions on how to use the tool. If you encounter any issues or have any questions when using PACE, please email PLOS at figures@plos.org. Please note that Supporting Information files do not need this step.
