# Supplementary material for: Inter-nesting area use, migratory routes, and foraging grounds for hawksbill turtles (Eretmochelys imbricata) in the Western Caribbean
Source: PLoS One. 2025 Mar 12;20(3):e0317778. doi: 10.1371/journal.pone.0317778 (PMC11902275; doi:10.1371/journal.pone.0317778)
Supplement: Attachment — Submitted filename: PONE-D-24-40236_reviewer Addison edit.docx [file pone.0317778.s001.docx]

**PLOS ONE**

**Inter-Nesting Area Use, Migratory Routes, and Foraging Grounds for Hawksbill Turtles (Eretmochelys imbricata) in the Western Caribbean**

--Manuscript Draft--

| **Manuscript Number:** | PONE-D-24-40236 |
| --- | --- |
| **Article Type:** | Research Article |
| **Full Title:** | Inter-Nesting Area Use, Migratory Routes, and Foraging Grounds for Hawksbill Turtles (Eretmochelys imbricata) in the Western Caribbean |
| **Short Title:** | Movements of nesting hawksbills in the Western Caribbean |
| **Corresponding Author:** | Stephen G. Dunbar Loma Linda University  Loma Linda, CA UNITED STATES OF AMERICA |
| **Keywords:** | satellite telemetry, ocean currents, cosine similarity, Honduras, Nicaragua, Costa Rica, Panama |
| **Abstract:** | The hawksbill turtle, Eretmochelys imbricata, has been at risk of extinction for more than 40 years and remains critically endangered. While nesting beach protection is important for hatchling production, identifying inter-nesting, migratory, and foraging habitats is crucial for mitigating threats to population recovery. We report the use of satellite telemetry to monitor movements of 15 hawksbill turtles in the Western Caribbean. Transmitters were deployed on nesting turtles in Honduras (2012 n = 2; 2017 n = 3), Costa Rica (2000 n = 2; 2014 n = 1; 2015 n = 1; 2021 n = 1; 2018 n = 4),  and Panama (2017 n = 1). Hawksbill inter-nesting habitats ranged from 4 - 2,643  km2 (core 50% utilization distribution) for the 15 – 70 tracking days. Large inter-nesting area use may be a result of habitats adjacent to a narrow continental shelf with strong ocean currents, causing turtles to actively search for suitable habitats. Following nesting, these turtles engaged in migrations to foraging grounds that covered 73 – 1,059 km lasting between 5 – 45 days. During migrations, turtles regularly altered their direction relative to ocean currents, using with-current movement to counteract against- current movement. Hawksbills from multiple beaches congregated in the same  foraging habitat, despite nesting in different years. Turtles in this study foraged along the coastal and continental shelves of Nicaragua, Honduras, Belize, and Mexico, with turtles from disparate nesting sites utilizing the Nicaragua Rise hotspot area. Foraging area use was generally smaller (n = 8, 6 – 705 km2) than inter-nesting area use, possibly indicating that foraging habitats provided necessary food and resting areas. These data help us better understand inter-nesting and foraging habitat locations, core area use, and post-nesting migrations. Together, this provides vital information to mitigate potential in-water threats to critically endangered adult hawksbills along Western Caribbean migration corridors. |
| **Order of Authors:** | Stephen G. Dunbar |
|  | Daniel R. Evans |
|  | Lindsey R. Eggers |
|  | Quintin D. Berman |
|  | Luis G. Fonseca |
|  | Frank V. Paladino |
|  | Lidia A. Salinas |
|  | Chelsea E. Durr |
| **Additional Information:** |  |
| **Question** | **Response** |
| **Financial Disclosure** | The author(s) received no specific funding for this work. |

| Enter a financial disclosure statement that describes the sources of funding for the work included in this submission. Review the [submission guidelines](http://journals.plos.org/plosone/s/submission-guidelines#loc-financial-disclosure-statement) for detailed requirements. View published research articles from [*PLOS ONE*](https://journals.plos.org/plosone/) for specific examples.  This statement is required for submission and **will appear in the published article** if the submission is accepted. Please make sure it is accurate. |  |
| --- | --- |
| **Funded studies**  Enter a statement with the following details:   - Initials of the authors who received each award - Grant numbers awarded to each author - The full name of each funder - URL of each funder website - Did the sponsors or funders play any role in the study design, data collection and analysis, decision to publish, or preparation of the manuscript? |  |
| Did you receive funding for this work? |  |
| **Competing Interests**  Use the instructions below to enter a competing interest statement for this submission. On behalf of all authors, disclose any [competing interests](http://journals.plos.org/plosone/s/competing-interests) that could be perceived to bias this work—acknowledging all financial support and any other relevant financial or non- financial competing interests.  This statement is required for submission and **will appear in the published article** if the submission is accepted. Please make sure it is accurate and that any funding sources listed in your Funding Information later in the submission form are also declared in your Financial Disclosure statement.  View published research articles from | The authors have declared that no competing interests exist. |

| [*PLOS ONE*](http://journals.plos.org/plosone/) for specific examples. |  |
| --- | --- |
| **NO authors have competing interests**  Enter: *The authors have declared that no competing interests exist.*  **Authors with competing interests**  Enter competing interest details beginning with this statement:  *I have read the journal's policy and the authors of this manuscript have the following competing interests: [insert competing interests here]* |  |
| * typeset |  |
| **Ethics Statement**  Enter an ethics statement for this submission. This statement is required if the study involved:   - Human participants - Human specimens or tissue - Vertebrate animals or cephalopods - Vertebrate embryos or tissues - Field research   Write "N/A" if the submission does not require an ethics statement.  General guidance is provided below. Consult the [submission guidelines](http://journals.plos.org/plosone/s/submission-guidelines#loc-guidelines-for-specific-study-types) for detailed instructions. **Make sure that all information entered here is included in the Methods section of the manuscript.** | This study was conducted in strict adherence to ethical conditions for the treatment and care of animals, and in compliance with national laws and permitted research. For Honduras, ethical approval was provided by the Loma Linda University IACUC protocol approval #s 89029 and 8150049 and Honduras national permit #s DGPA-005-2006 and DGPA-245-2006; for Costa Rica, Perdue University IACUC# 1206000656 and permit #s ACLAC-314-2018, ACLAC-315-2018, ACLAC-316-2018, ACLAC-317-2018, ACLAC-318-2018, and SINAC-ACTo-DIR-PI-RES-003-2024; for Panama, SE/A-56- 2017. |

| **Format for specific study types**  **Human Subject Research (involving human participants and/or tissue)**   - Give the name of the institutional review board or ethics committee that approved the study - Include the approval number and/or a statement indicating approval of this research - Indicate the form of consent obtained (written/oral) or the reason that consent was not obtained (e.g. the data were analyzed anonymously)   **Animal Research (involving vertebrate animals, embryos or tissues)**   - Provide the name of the Institutional Animal Care and Use Committee (IACUC) or other relevant ethics board that reviewed the study protocol, and indicate whether they approved this research or granted a formal waiver of ethical approval - Include an approval number if one was obtained - If the study involved *non-human primates*, add *additional details* about animal welfare and steps taken to ameliorate suffering - If anesthesia, euthanasia, or any kind of animal sacrifice is part of the study, include briefly which substances and/or methods were applied   **Field Research**  Include the following details if this study involves the collection of plant, animal, or other materials from a natural setting:   - Field permit number - Name of the institution or relevant body that granted permission |  |
| --- | --- |
|  |  |
| **Data Availability**  Authors are required to make all data underlying the findings described fully available, without restriction, and from the time of publication. PLOS allows rare exceptions to address legal and ethical concerns. See the [PLOS Data Policy](http://journals.plos.org/plosone/s/data-availability) and [FAQ](http://journals.plos.org/plosone/s/data-availability#loc-faqs-for-data-policy) for detailed information. | No - some restrictions will apply |

| A Data Availability Statement describing where the data can be found is required at submission. Your answers to this question constitute the Data Availability Statement and **will be published in the article**, if accepted. |  |
| --- | --- |
| **Important:** Stating ‘data available on request from the author’ is not sufficient. If your data are only available upon request, select ‘No’ for the first question and explain your exceptional situation in the text box. |  |
| Do the authors confirm that all data underlying the findings described in their manuscript are fully available without restriction? |  |
| **Describe where the data may be found in full sentences. If you are copying our sample text, replace any instances of XXX with the appropriate details.**   - If the data are **held or will be held in a public repository**, include URLs, accession numbers or DOIs. If this information will only be available after acceptance, indicate this by ticking the box below. For example: *All XXX files are available from the XXX database (accession number(s) XXX, XXX.*). - If the data are all contained **within the manuscript and/or Supporting Information files**, enter the following: *All relevant data are within the manuscript and its Supporting Information files.* - If neither of these applies but you are able to provide **details of access elsewhere**, with or without limitations, please do so. For example:   *Data cannot be shared publicly because of [XXX]. Data are available from the XXX Institutional Data Access / Ethics Committee (contact via XXX) for researchers who meet the criteria for access to confidential data.*  *The data underlying the results presented in the study are available from (include the name of the third party* | Data cannot be shared publicly because we intend to continue to analyze other dimensions of the data for relevant aspects of sea turtle behavior in additional manuscripts. |

| *and contact information or URL).*   - This text is appropriate if the data are owned by a third party and authors do not have permission to share the data.   * typeset |  |
| --- | --- |
| Additional data availability information: |  |

Manuscript	[Click here to access/download;Manuscript;Dunbar et al_HawksbillTelemetrySub.pdf](https://www2.cloud.editorialmanager.com/pone/download.aspx?id=37544450&guid=e48ab469-4046-4c66-9ea1-1d2e0e8825ba&scheme=1)

### 1 Full title: Inter-nesting area use, migratory routes, and foraging grounds for

2 hawksbill turtles (*Eretmochelys imbricata*) in the Western Caribbean

### 3 Short title: Movements of nesting hawksbills in the Western Caribbean

4 Stephen G. Dunbar^1,2,3,*^, Daniel R. Evans^4^, Lindsey R. Eggers^5^, Quintin D. Bergman^6^, Luis G.
5 Fonseca^7^, Frank V. Paladino^6,8^, Lidia Salinas^2,3^, Chelsea E. Durr^6,8^
6 ^1^Marine Research Group, Loma Linda University, Loma Linda, CA, 92350 United States of
7 America
8 ^2^Protective Turtle Ecology Center for Training, Outreach, and Research, Inc. (ProTECTOR,
9 Inc.), Loma Linda, CA, 92350 United States of America
10 ^3^Protective Turtle Ecology Center for Training, Outreach, and Research, Inc. (ProTECTOR -
11 Honduras), Tegucigalpa, 11101 Honduras
12 ^4^Sea Turtle Conservancy, Gainesville, FL, 32609 United States of America
13 ^5^Seattle Aquarium, Seattle, WA, 98101 United States of America
14 ^6^Department of Biology, Purdue University Fort Wayne, Fort Wayne, IN, 46805 United States of
15 America
16 ^7^Biocenosis Marina, Trinidad de Moravia, San José, 11403 Costa Rica
17 ^8^The Leatherback Trust, Fort Wayne, IN, 46835 United States of America
18 *****Corresponding author
19 E-mail: [sdunbar@llu.edu](mailto:sdunbar@llu.edu) (SD)

# 20 ABSTRACT

21 The hawksbill turtle, *Eretmochelys imbricata*, has been at risk of extinction for more than
22 40 years and remains critically endangered. While nesting beach protection is important for
23 hatchling production, identifying inter-nesting, migratory, and foraging habitats is crucial for
24 mitigating threats to population recovery. We report the use of satellite telemetry to monitor
25 movements of 15 hawksbill turtles in the Western Caribbean. Transmitters were deployed on
26 nesting turtles in Honduras (2012 n = 2; 2017 n = 3), Costa Rica (2000 n = 2; 2014 n = 1; 2015 n

27	= 1; 2021 n = 1; 2018 n = 4), and Panama (2017 n = 1). Hawksbill inter-nesting habitats ranged

28 from 4 - 2,643 km2 (core 50% utilization distribution) for the 15 – 70 tracking days. Large inter-
29 nesting area use may be a result of habitats adjacent to a narrow continental shelf with strong
30 ocean currents, causing turtles to actively search for suitable habitats. Following nesting, these
31 turtles engaged in migrations to foraging grounds that covered 73 – 1,059 km lasting between 5 –
32 45 days. During migrations, turtles regularly altered their direction relative to ocean currents,
33 using with-current movement to counteract against-current movement. Hawksbills from multiple
34 beaches congregated in the same foraging habitat, despite nesting in different years. Turtles in
35 this study foraged along the coastal and continental shelves of Nicaragua, Honduras, Belize, and
36 Mexico, with turtles from disparate nesting sites utilizing the Nicaragua Rise hotspot area.
37 Foraging area use was generally smaller (n = 8, 6 – 705 km^2^) than inter-nesting area use,
38 possibly indicating that foraging habitats provided necessary food and resting areas. These data
39 help us better understand inter-nesting and foraging habitat locations, core area use, and post-
40 nesting migrations. Together, this provides vital information to mitigate potential in-water threats
41 to critically endangered adult hawksbills along Western Caribbean migration corridors.

# 42 Introduction

43 The hawksbill sea turtle, *Eretmochelys imbricata*, can be found in tropical waters around the
44 globe (1). This species has been exploited by humans for centuries for its meat and eggs,
45 although direct take was primarily due to its elaborately colored carapace which is rendered into
46 tortoiseshell products and sold internationally (2-5). Trade in tortoiseshell was and is a primary
47 cause of the severe decline in hawksbill populations worldwide (5, 6). Due to substantial
48 decreases in numbers, the hawksbill turtle is currently listed as Critically Endangered on the
49 International Union for Conservation of Nature (IUCN) *Red List of Threatened Species* (7).
50 While addressing the illegal tortoiseshell trade and implementing nesting beach protections are
51 vital to conservation efforts for the species, these alone are insufficient to restore populations. A
52 holistic approach to conservation, in which every life stage is addressed, is necessary if sea turtle
53 populations are to recover (8-11). Many sea turtle species, including hawksbills, migrate between
54 foraging grounds and nesting sites, with studies indicating sea turtles may travel along
55 established migratory corridors between the two. For example, Pendoley et al. (12) found fidelity
56 to migrating corridors in flatbacks (*Natator depressus*) off the northwest coast of Australia, while
57 the same behavior was reported by Marcovaldi et al. (13) in migrating loggerheads (*Caretta*
58 *caretta*) in waters off northwestern Brazil. The existence of migratory corridors for specific turtle
59 populations provides opportunities for protected species managers to take steps to protect turtles
60 at both national and multinational scales.
61 Of the seven extant sea turtle species, the migration and movement patterns of hawksbills are the
62 least well known (14). Initially, researchers suggested that hawksbills did not embark on
63 extensive migrations (1, 15, 16). We now understand through both genetic analyses and satellite
64 telemetry, that nesting hawksbills at a given site may aggregate from foraging grounds located in
65 many different countries. For example, Meylan (17) collected tag return data on hawksbills in the
66 Caribbean indicating that turtles were migrating from their nesting grounds in Tortuguero, Costa
67 Rica, to a variety of countries, including Nicaragua, Honduras, and Panama. Troëng et al. (18)
68 utilized flipper tag returns as well as satellite telemetry to document hawksbill migrations from
69 Tortuguero, Costa Rica, to waters off Nicaragua and Honduras, including the Honduran island of
70 Guanaja in the Bay Islands. A study by van Dam et al. (19) followed hawksbills nesting on Mona
71 Island, Puerto Rico, and documented their journeys to the French West Indies, Nicaragua,
72 Honduras, Turks and Caicos, the U.S. Virgin Islands, and the British Virgin Islands. Turtles have
73 been found more than 2,000 km from where they were tagged and up to 15 years after they were
74 tagged, indicating that hawksbill turtles are capable of the same dispersal patterns as other sea
75 turtle species (19, 20). Satellite telemetry has been invaluable in addressing gaps in our
76 knowledge with regard to at-sea movements and behavior of sea turtles, and while the data is
77 plentiful for some species, sample sizes remain small particularly for hawksbills (21-23).
78 Therefore, we used satellite telemetry to identify the movements and habitat use of hawksbill
79 turtles in the Western Caribbean to expand our knowledge of this imperiled species. Historically,
80 extensive nesting populations of hawksbills were present throughout the Caribbean (24). The
81 Bay Islands off the north coast of Honduras; Tortuguero, Costa Rica; and the Bocas del Toro
82 Province of Panama are all areas that were originally productive hawksbill nesting areas (6, 25-
83 27). In Costa Rica, hawksbill populations saw a more than 77% decline between the 1950s and
84 the early 2000s (18). Similarly, in Panama, by the 1990s best estimates indicated that the
85 populations had declined by almost 98% (6). Some aspects of these declines have been addressed
86 by the standard terrestrial and coastal protections. For example, Costa Rica has 166 protected
87 areas that encompass 50% of the country’s coastline, 20 of which are Marine Protected Areas
88 (MPA) (28). One of these is Tortuguero National Park, established in 1975 to protect nesting
89 turtles, nesting beaches, and adjacent terrestrial habitats. Another is an MPA that is incorporated
90 in the Gandoca-Manzanillo Wildlife Refuge (GMNWR) which is included in the Area
91 Conservación de la Amistad Caribe, located in southeast Costa Rica. Hawksbills are known to
92 make nesting visits to the larger beach, Playa Gandoca, and the smaller beach, Playita, both of
93 which are located within GMNWR (29). However, this MPA was designed without specific
94 knowledge of hawksbill spatial ecology; therefore, the GMNWR may lack sufficient protection
95 of hawksbill habitats. While these coastal protections have varying degrees of success (i.e. more
96 if they were informed by sea turtle research and potentially less if they were not), there is also
97 need to address protection of migration routes and foraging habitats. These are more challenging
98 because they may not be within any one country, because they are in
99 international waters (30). Known foraging habitats in the Western Caribbean are located along
100 the continental shelves of Nicaragua and Honduras, as well as Belize and Mexico (14, 18, 30-
101 35). This is because hawksbill foraging requires coastal waters where potential prey items such
102 as sponges, tunicates, corals, or algae (36-39) occur in sufficient abundance. These habitats
103 provide important opportunities, yet require significant understanding of turtle use, in order to
104 implement important conservation measures. Nevertheless, many threats to hawksbill turtles
105 remain in the form of fisheries bycatch, direct intentional take of turtles in-water and at nesting
106 beaches, and the loss of habitat due to degradation and coastal development with few regulatory
107 restrictions.
108 In this study, we used satellite telemetry to 1) measure utilization distribution of area-restricted
109 search behavior adjacent to nesting beaches (i.e. inter-nesting (IN) area); 2) define migratory
110 corridors connecting nesting beaches to foraging grounds; 3) identify foraging habitats and
111 quantify utilization distribution of area-restricted search behavior therein; and 4) calculate cosine
112 similarity between direction of turtle movements in relation to ocean current directions.

# 113 Methods

114 This study was conducted in strict adherence to ethical conditions for the treatment and care of
115 animals, and in compliance with national laws and permitted research. For Honduras, ethical
116 approval was provided by the Loma Linda University IACUC protocol approvals #89029
117 and **#**8150049 and Honduras national permits DGPA-005-2006 and DGPA-245-2006; for Costa
118 Rica, Perdue University IACUC #1206000656 and permit numbers ACLAC-314-2018, ACLAC-

119	315-2018, ACLAC-316-2018, ACLAC-317-2018, ACLAC-318-2018, and SINAC-ACTo-DIR-

120 PI-RES-003-2024; for Panama, permit SE/A-56-2017.

## 121 Nesting beach/transmitter deployment sites

122 We deployed satellite transmitters on 15 hawksbill sea turtles from four beaches in the Western
123 Caribbean: Pumpkin Hill Beach (PHB), Utila, Honduras (PHB, Fig 1A); Tortuguero National
124 Park, Costa Rica (TNP, Fig 1B); Gandoca-Manzanillo National Wildlife Refuge, Costa Rica
125 (GMNWR, Fig 1C); and Chiriqui Beach, Panama (CBP; Fig 1D).
126 Fig 1. Study site map including the locations of the beaches where we deployed satellite
127 transmitters on nesting hawksbill sea turtles (*Eretmochelys imbricata*). We deployed transmitters
128 on A) Utila, Honduras in 2012 (n = 2) and 2017 (n = 3); B) Tortuguero, Costa Rica in 2000 (n =
129 2), 2014 (n = 1), 2015 (n = 1), and 2021 (n = 1); C) Gandoca-Manzanillo National Wildlife
130 Refuge, Costa Rica in 2018 (n = 4); and D) Chiriqui Beach, Panama in 2017 (n = 1). Map was
131 made in ArcGIS Pro using GADM shapefiles (https://gadm.org/index.htm).
132 Utila is the smallest of the three Bay Islands in northern Honduras and is located approximately
133 35 km off the mainland. The Bay Islands lie at the southern end of the Mesoamerican Barrier
134 Reef System, which extends north along the coasts of Guatemala, Belize, and Mexico,
135 terminating at the tip of the Yucatan Peninsula. The island of Utila is 11 km long and 5 km wide,
136 with two-thirds of the island covered by swamp and mangrove forests. Fourteen beaches
137 (including nearby cays) have been assessed as potential nesting beaches for hawksbills (40), yet
138 only two of these beaches have documented regular nesting. One is PHB (16° 07’ N, 86° 53’ W),
139 where this study took place during the 2012 and 2017 nesting seasons, in conjunction with
140 annual beach monitoring conducted jointly by ProTECTOR Inc., and the Bay Islands
141 Conservation Association (BICA) Utila (41). During the months of June through October,
142 surveillance procedures involved walking the beach nightly from 18:00 to 04:30 hours. We
143 walked the length of the beach once per hour, as Stapleton and Eckert (42) determined that
144 hourly patrols are likely to encounter almost all nesting turtles. Two nesting hawksbills in 2012
145 and three nesting hawksbills in 2017 encountered during beach monitoring were selected for
146 platform terminal transmitter (PTT) application.
147 Tortuguero National Park, Costa Rica (10° 33' N, 83° 30' W) was established in 1975 to protect
148 the largest green turtle rookery in the Western Hemisphere. The park includes over 19,000
149 hectares (46,900 acres) and protects 33.8 km of black sand nesting beach from the mouth of the
150 Tortuguero River south to the river mouth at Jalova, just north of Parismina, Costa Rica. In
151 addition to green turtles (*Chelonia mydas*), both leatherback (*Dermochelys coriacea*) and
152 hawksbill turtles also nest in TNP. During the months of June through October, the northern 8
153 km of beach within the TNP were patrolled by the Sea Turtle Conservancy on foot nightly from
154 20:00 to 04:00 hours. The beach was patrolled each night during the entire time period to
155 encounter nesting turtles. We flipper-tagged nesting turtles, assessed turtle health conditions,
156 recorded carapace measurements, and counted the number of eggs deposited. Two nesting
157 hawksbills in 2000, one in 2014, one in 2015, and one in 2021 were encountered during beach
158 monitoring and selected for PTT application.
159 Gandoca-Manzanillo National Wildlife Refuge, Costa Rica (09° 37’ N, 82° 40’ W) consists of
160 50.13 km2 of terrestrial area and 44.36 km2 of marine area for a total of 94.49 km2 of protected
161 habitat (43). This includes ?? km of beach that supports sea turtle nesting. Listed as a Ramsar site by the RAMSAR Convention on Wetlands of International
162 Importance, GMNWR is a coastal lagoon consisting of seagrass beds, coral reefs, beaches, and
163 cliffs with flooded lowland areas (44). Anthropogenic uses in the area include traditional, low-
164 scale agriculture growing cereals, cacao, plantains, yucca, and other tuberous plants; forestry;
165 and marine and freshwater fishing (44). Large-scale banana cultivation also occurs in the area
166 adjacent to the reserve and communities (Q.D.B. personal observation). Created to protect
167 endangered species and to maintain them in their natural habitat, GMNWR supports a high
168 diversity of species including birds, reptiles, mollusks, fish (marine, estuarine, and freshwater),
169 crustaceans (including lobsters), and 32 coral species (44). Four of the five sea turtles of the
170 Caribbean nest in GMNWR including the loggerhead (*Caretta caretta*), green, leatherback, and
171 hawksbill turtles (29, 45).
172 Chiriquí Beach, Panama (08° 56’ N, 81° 39’ W), in the Comarca Ngöbe-Buglé, is an important
173 Caribbean hawksbill turtle rookery, as well as the most important nesting beach for leatherbacks
174 in Caribbean Central America. Chiriquí Beach represents 22 km of beach along the Caribbean
175 Coast of Panama. Starting in 2003, Sea Turtle Conservancy began a sustained presence
176 monitoring Chiriquí Beach. Night patrols were conducted during the months of March through
177 September for 6 hours each night to observe and flipper tag females. In addition, we collected
178 data for each turtle including time, activity at first encounter, carapace measurements, presence
179 of tag scars or overgrown tags, and any mutilations or deformities. One nesting hawksbill in
180 2017 was selected for PTT application.

## 181 Study animals

182 All of the turtles tracked during this study were nesting female hawksbills with no apparent
183 deformities (e.g., missing limbs, carapace malformations). Prior to transmitter deployment, we
184 tagged the turtle with a unique identifying tag; either a Passive Integrated Transponder (PIT) tag
185 (GMNWR) and/or metal Inconel 651-style tags, if one was not already present (PHB, TNP,
186 CBP). When used, PIT tags were injected into the shoulder just under the skin, whereas metal
187 Inconel tags were attached to the right front and/or right rear flippers, with tags placed on the
188 proximal scale on the trailing edge of the flippers as per Dunbar and Berube (46) and Damazo
189 (41). Finally, we measured curved carapace length (CCL) and curved carapace width (CCW).

## 190 PTT Attachment

191 All transmitters were carapace-mounted transmitters generally following procedures from other
192 transmitter studies (19, 47, 48) (Table 1). We cleaned the carapace using a combination of water,
193 steel wool, sponges, sandpaper, and then isopropyl alcohol. The final step involved cross-
194 hatching/scoring the second central scute to help the fastener adhere to the shell. At PHB, SPOT
195 5 and SPOT 293A (Wildlife Computers, Redmond, WA, USA) transmitters were attached using
196 Sika Anchorfix two-part epoxy. At TNP, the ST-23 transmitters (Telonics, Mesa, AZ, USA)
197 were attached using fiberglass cloth and resin with an added roll of Kevlar fiberglass anterior to
198 the base of the antenna as additional protection for the antenna. The KiwiSat 202 (Sirtrack/Lotek,
199 Seattle, WA, USA) and SPOT 5 tags were attached using a two-part epoxy (Powers 308+,
200 Powers Fasteners, Brewster, NY). Finally, the SPLASH10 was attached using the Wildlife
201 Computers Sea Turtle Attachment Method (49) consisting of an epoxy putty, resin, and
202 fiberglass. At GMNWR and CBP, KiwiSat 202 transmitters were attached using a two-part
203 Pure50+ epoxy (Powers Fasteners, Brewster, NY), and two-part powers 308+ epoxy (Powers
204 Fasteners, Brewster, NY), respectively. In all cases, this process took between 30 and 120
205 minutes and turtles were immediately permitted to return to the water, except at TNP, where
206 turtles were kept in boxes overnight to have transmitters attached in the morning.
207 Table 1. Data on transmitter attachment location, date, transmitter type, and area restricted search (ARS)
208 as defined by the switching state-space model. ARS location points were then used to calculate the area
209 (km^2^) of the 50% and 95% utilization distribution (UD). PHB = Pumpkin Hill Beach, Utila, Honduras;
210 TNP = Tortuguero National Park, Costa Rica; GMNWR = Gandoca-Manzanillo National Wildlife
211 Refuge, Costa Rica; CBP = Chiriqui Beach, Panama.

Inter-Nesting Area (km^2^)	Foraging A

Deploy	Transmitter

Deployment Date	Transmitter Type	Inter-Nesting

Foraging

Location	ID

|  | | | 50% UD | 95% UD |  | 50% UD |
| --- | --- | --- | --- | --- | --- | --- |
| 108759 | Jul 11, 2012 | 15 | 53 | 211 | 63 | 31 |
| 108760 | Aug 12, 2012 | - | - | - | 15 | 8 |

ARS (d)

ARS (d)

PHB

| 119995 | Sep 13, 2017 | Wildlife Computers SPOT | - | - | - | 59 | 6 |
| --- | --- | --- | --- | --- | --- | --- | --- |
| 120378 | Sep 13, 2017 |  | 5* | 26 | 98 | 57 | 441 |
| 138816 | Sep 14, 2017 |  | 5* | 47 | 233 | 54 | 7 |
| 22126  22134 | Jul 25, 2000  Jul 26, 2000 | Telonics ST-23 | 24  - | 1,234  - | 6,697  - | 97  436 | 128  151 |

TNP

137659	Jul 4, 2014	SirTrack KiwiSat

202

129347	Jul 2, 2015	Wildlife Computers SPOT

70	888	3,852	20	137

| 47 | 664 | 2,360 | 598 | 14 |
| --- | --- | --- | --- | --- |
| 68 | 254 | 1,055 | - | - |

220791	Jul 13, 2021

Wildlife Computers SPLASH 10

21	2,643	12,315	41	705

GMNWR

107904	Aug 24, 2018	SirTrack KiwiSat

202

55	201	1,170	276	242

107915	Aug 26, 2018	12	17	86	784	23

202

|  | | - | - | - | 414 | 27 |
| --- | --- | --- | --- | --- | --- | --- |
| 107910 | Aug 28, 2018 | 42 | 213 | 1,301 | 315 | 15 |
| 107913 | Aug 29, 2018 | - | - | - | 472 | 28 |
| CBP 160923 May 30, 2017 SirTrack KiwiSat 65 | | | 4 | 24 | 472 | 6 |

212 *These turtles did not display inter-nesting behavior because 5 days is not sufficient to nest a subsequent
213 time. These represent the days the turtle spent in the vicinity of the nesting beach prior to migrating.

## 214 Data analysis

215 We downloaded location data relayed via the Argos satellite system and removed any points that
216 were on land. We then applied the Freitas et al. (50) location filter (sdafilter, argosfilter package)
217 in R (R statistical software, R Version 4.3.1, Vienna, Austria). We removed locations requiring a
218 max velocity of > 1.389 m/s and locations requiring a turn angle < 12° with a corresponding
219 spike distance of 2,500 m or those requiring < 25° with a corresponding spike distance of 5,000
220 m (51-53). Subsequently, a Bayesian state-space model was fitted to each set of filtered turtle
221 location data individually using the “bsam” package in R (54-56). Here, we used the switching
222 correlated random walk model which estimates both location and behavioral states. Parameters
223 for our model were a time step of one day, 10,000 Markov Chain Monte Carlo samples for
224 adaptation and burn-in, 5,000 samples generated after burn-in, every 10th sample was retained to
225 reduce autocorrelation, and a smoothing value of 0.3 was used. This model returned a behavioral
226 mode of either 1 or 2, where 1 is considered transitioning (migrating) behavior and 2 is
227 considered area restricted search (ARS) behavior, which is usually associated with either inter-
228 nesting (when ARS is located adjacent to the turtle’s nesting beach) or foraging behavior (when
229 ARS is located in oceanic habitats or those removed from known nesting beaches). This divided
230 our data into two behavioral states. We used the “adehabitathr” package in R (57, 58) to estimate
231 kernel home range with 50% and 95% utilization distribution (UD) contours to represent core
232 and resident areas, respectively, and generate shapefiles. Finally, for each track segment, defined
233 as the straight-line distance between two consecutive location points (A and B), we calculated
234 the direction. To do this, we used the Haversine equation to estimate the track length between the
235 two points (A and B) and the east-west distance:

2 φ𝐵 − φ𝐴

2 λB − λA

𝑎 = 𝑠𝑖𝑛 (

) + 𝑐𝑜𝑠 φA ⋅ 𝑐𝑜𝑠 φB ⋅ 𝑠𝑖 (	)

2	2

237	𝑐 = 2 ⋅ 𝑎𝑡𝑎𝑛2(√𝑎, √1 − 𝑎)

238 distance = 𝑅 ⋅ 𝑐
239 where *φ* is the latitude, *λ* is the longitude, atan2 measures the counterclockwise angle *θ*, in
240 radians, between the positive x-axis and the point, and R is earth’s radius (mean radius = 6,371
241 km). Together these segments allowed us to calculate the angle from due East.
242 In ArcGIS Pro (Version 3.2.1, 2023, Esri, Redlands, CA, USA), we mapped individual tracks,
243 and calculated UD area in ArcGIS from shapefiles and spatial data (country shapefiles:
244 <https://gadm.org/index.html>; bathymetry shapefile: <https://www.gebco.net/>; and economic
245 exclusion zones shapefile: [www.marineregions.org](http://www.marineregions.org/)). We downloaded temporally relevant
246 historical satellite data for this project due to the historical nature of the turtle movements. Ocean
247 current direction was calculated by using data from Copernicus Marine Services
248 (<https://marine.copernicus.eu/>) and ArcGIS Pro to transform vector information into the angle
249 from due East. In this way, we could calculate the cosine similarity *SC* between the two angles
250 using:

251	𝑆(θ1, θ2) = 𝑐𝑜𝑠(θ1 − θ2)

252 where θ1 and θ2 are angles, calculated from due East, for the hawksbill migration track and ocean
253 current movement at the track segment, respectively. An angle close to 0° or 360° indicates a
254 direction of movement that is east, while an angle of approximately 180° indicates a westward
255 movement direction. An *SC* of −1 means the movement direction of the turtle is directly against
256 the direction of ocean current movement. An *SC* of 1 indicates that the turtle is moving in the
257 same direction as the ocean currents. We then weighted the *SC* by the track length.

# 258 Results

## 259 Inter-nesting and nest adjacent ARS

260 We deployed satellite transmitters on 15 hawksbill turtles between 2000 and 2021 (Table 1).
261 Turtles were tracked between 22 and 1,254 days, spending between 0 and 70 IN days in the
262 vicinity of the nesting beach before engaging in post-nesting migrations.
263 In Honduras, five turtles were tracked from PHB. Post-tagging, turtle 108759 engaged in inter-
264 nesting behavior for 15 days and had a core UD of 53 km2 before nesting again (Table 1). Turtles
265 120378 and 138816 each spent 5 days in the area of Utila (core UDs of 26 and 47 km^2^,
266 respectively) before engaging in post-nesting migrations (Fig 2A). For these three turtles, ARS
267 was associated with the southern side of the island where water was less than 100 m deep. The
268 final two turtles, 108760 and 119995, began their post-nesting migrations immediately.
269 Fig 2. Inter-nesting area restricted search for core (50%) utilization distribution of hawksbill sea
270 turtles nesting on: (A) Pumpkin Hill Beach, Utila, Honduras (pink); (B) Tortuguero, Costa Rica
271 (orange); (C) Gandoca-Manzanillo National Wildlife Refuge, Costa Rica (red); and (D) Chiriqui
272 Beach, Panama (brown). Map was made in ArcGIS Pro using GADM shapefiles
273 (https://gadm.org/index.htm) and GEBCO bathymetry. For Chiriqui Beach, we included both
274 core and resident (95%) contour lines.
275 At TNP, turtles 22126, 137659, 129347, and 220791 engaged in inter-nesting ARS from between
276 21 to 70 days, suggesting that each of these turtles likely nested at least one subsequent time, in
277 some cases possibly more (Fig 2B). Turtle 22126 engaged in inter-nesting behavior for 24 days
278 and had a core area of 1,234 km2 (Table 1). For turtle 137659, we recorded two inter-nesting
279 seasons, one in 2014 that lasted 47 days (50% UD: 664 km^2^) when the transmitter was deployed
280 and one in 2016 (50% UD: 254 km^2^) after re-migration, which lasted 68 days until transmissions
281 stopped. Turtle 129347 spent 70 inter-nesting days in a core area of 888 km^2^, and turtle 220791
282 remained near Tortuguero for 21 days in a core area of 2,643 km2 (Table 1, Fig 2B). Overall,
283 core IN area use was 1,357 ± 889 km^2^.
284 At the GMNWR, Costa Rica, four hawksbill turtles were tracked starting in August 2018. Three
285 turtles (107904, 107915, 107910) engaged in inter-nesting behavior that lasted between 13, 28,
286 and 56 days and had core UDs of 17, 213, and 201 km^2^, respectively (Table 1, Fig 2C). Turtles
287 107904 and 107915 remained near the nesting beach, while turtle 107910’s IN duration (42 d)
288 was split between the nesting beach area and Bocas del Toro, Panama. It is likely that these
289 turtles each nested a subsequent time(s). Taken together, turtles at GMNWR had a mean core IN

290	area of 144 ± 110 km^2^.

291 At CBP, a single turtle (160923) was tagged in 2021. This turtle spent 65 d in a coastal inter-
292 nesting habitat north of the nesting beach, with this amount of time sufficient to nest multiple
293 times (Table 1, Fig 2D). Core UD was 4 km2 and residential UD was 24 km2 (Table 1).

## 294 Migration

295 Migrations lasted 5 to 46 days (mean = 22 ± 12 d SD) and turtles traveled between 73 and 1,522
296 km (mean = 585 ± 433 km SD), remaining south of the Caribbean Current. Turtles displayed an
297 average calculated migration travel speed of 26 ± 9.5 km/d (7 – 43 km/d), and preferred coastal
298 and continental shelf foraging in Nicaragua, Honduras, Belize, and Mexico where water was

shallow and water currents were slower (Fig 3).

301 Fig 3. Movements and area use of hawksbill turtles in relation to nesting beaches and
302 bathymetry. Turtle tracks in pink were tagged on Pumpkin Hill Beach, Utila, Honduras (PHB);
303 orange tracks were from turtles tagged at Tortuguero, Costa Rica (TNP); red tracks were from
304 turtles tagged on Gandoca-Manzanillo National Wildlife Refuge, Costa Rica (GMNWR); and the
305 brown track was from a turtle tagged on Chiriqui Beach, Panama (CBP). Deployment dates can
306 be found in Table 1. This map was made using bathymetry from GEBCO, and the map was made
307 using ArcGIS Pro.
308 Turtles nesting on PHB engaged in migrations that lasted between 5 and 33 days and covered
309 between 73 and 434 km track distance (Table 2). Turtle 108759 began her post-nesting migration
310 by traveling from Utila in a northwesterly direction, reaching the mainland of Belize at
311 Ambergris Caye (Fig 4). From here, the turtle traveled north along the coast until reaching a
312 foraging habitat in Bahia de la Ascension, Mexico (Fig 4). The second turtle tagged in August
313 2012 (108760) departed the nesting area immediately, and traveled 202 km in 8 days to reach
314 Middle Long Caye, Belize (Table 2, Fig 4). Of the three turtles tagged in 2017, 119995 departed
315 directly, while turtles 120378 and 138816 spent 5 days around Utila before migrating. Turtle
316 119995 traveled 73 km in 5 days and began foraging approximately 40 km off the west coast of
317 Utila (Table 2, Fig 4). Turtle 120378 traveled 237 km in 33 days. During the initial 5 days, the
318 turtle crossed over open ocean and skirted the south end of Glover’s Reef, then headed southwest
319 to the waters off of the city of Placencia. After this, the turtle engaged in a wandering style
320 behavior through the cays, finally settling east of Laughing Bird Caye, Belize. This wandering
321 behavior was included in both the migration map (Fig 4) and the foraging UD (Table 2). Finally,
322 turtle 138816 traveled 243 km in 13 days. After 7 days, the turtle reached the cays at the south
323 end of Glover’s Reef Atoll at which point she migrated north in the sheltered water, ending
324 between Drowned Cayes (Middle Long Caye)/Turneffe Atoll and mainland Belize (Belize City,
325 Fig 4).
326 Table 2. Migration data for hawksbill turtles nesting in the Western Caribbean, including nesting
327 beach/tagging location, migration dates, migration track length, average travel speed, average travel
328 direction in degrees from due east, average ocean current direction in degrees from due east, and the
329 average daily cosine similarity (weighted *S_C_*) between the direction of turtle movement and the direction
330 of water movement, weighted by the daily track length. Migration track lengths were calculated using the
331 Haversine formula. PHB = Pumpkin Hill Beach, Utila, Honduras; TNP = Tortuguero National Park,
332 Costa Rica; GMNWR = Gandoca-Manzanillo National Wildlife Refuge, Costa Rica; CBP = Chiriqui
333 Beach, Panama.

Beach	Tag	Migration Dates	Migration (d)	Track Length Speed (km/d)	Track

|  | | | | (km) |  | Direction |
| --- | --- | --- | --- | --- | --- | --- |
|  | 108759 | July 25 – August 7, 2012 | 14 | 433.89 | 30.99 | 176.92 |
|  | 108760 | August 13 – August 20, 2012 | 8 | 201.97 | 25.25 | 121.27 |
| PHB | 119995 | September 14 – September 18, 2017 | 5 | 72.92 | 14.58 | 176.22 |
|  | 120378 | September 18 – October 20, 2017 | 33 | 237.46 | 7.20 | 191.93 |
|  | 138816 | September 15 – September 27, 2017 | 13 | 242.50 | 18.65 | 132.57 |
|  | 22126 | August 17 – September 14, 2000 | 29 | 426.21 | 14.70 | 109.71 |
|  | 22134 | July 29 – August 14, 2000 | 17 | 300.62 | 17.68 | 103.51 |
| TNP | 137659 | August 21 – September 11, 2014 | 22 | 619.33 | 28.15 | 79.91 |
|  |  | May 1 – May 15, 2016 | 16 | 465.02 | 29.06 | 258.10 |
|  | 129347 | September 10 - September 26, 2015 | 17 | 363.37 | 21.37 | 117.12 |

|  | | 220791 | August 2 – August 20, 2021 | 19 | 498.74 | 26.25 | 73.27 |
| --- | --- | --- | --- | --- | --- | --- | --- |
|  |  | 107904 | October 17 – November 11, 2018 | 26 | 969.75 | 37.30 | 87.32 |
|  |  | 107915 | September 6 – October 17, 2018 | 44 | 1,521.69 | 34.58 | 138.37 |
|  | GMNWR | 107910 | October 09 – October 21, 2018 | 14 | 604.87 | 43.21 | 85.44 |
|  |  | 107913 | August 29 – October 13, 2018 | 46 | 1,493.98 | 32.48 | 111.58 |
|  | CBP | 160923 | August 3 – September 1, 2017 | 30 | 923.57 | 30.79 | 93.05 |
| 334 |  |  |  |  |  |  |  |

335	Fig 4. Migrations of hawksbill sea turtles nesting on Pumpkin Hill Beach, Utila, Honduras.

336	Turtles 108759 and 108760 were tagged in 2012; turtles 120378, 138816, and 119995 were

337 tagged in 2017. Map was made in ArcGIS Pro using GADM shapefiles
338 (https://gadm.org/index.htm) and GEBCO bathymetry.
339 Turtles nesting at TNP engaged in coastal migratory routes traveling between 300 and 620 km
340 over 16 to 29 days, reaching foraging grounds between central and northern Nicaragua (Table 2,
341 Fig 5). Turtles 22126 and 22134 traveled 426 and 301 km, respectively, in 29 and 17 days (Table
342 2). Turtle 137659 migrated 620 km in 22 days to reach the continental shelf off Northern
343 Nicaragua, then, in 2016, remigrated 465 km in 16 days to return to TNP (Fig 6). Turtle 129347,
344 tagged in 2015, migrated 363 km ending on the continental shelf of central Nicaragua (Table 2,
345 Fig 5). Turtle 220791 traveled 499 km in 19 days to forage on Half Moon Reef, on the
346 continental shelf adjacent to the border of Nicaragua and Honduras (Table 2; Fig 5).
347 Fig 5. Post-nesting migrations of hawksbill sea turtles nesting at Tortuguero, Costa Rica. Turtles
348 22126 and 22134 were tagged in 2000, turtle 137659 was tagged in 2014, turtle 129347 was
349 tagged in 2015, and turtle 220791 was tagged in 2021. Map was made in ArcGIS Pro using
350 GADM shapefiles (https://gadm.org/index.htm) and GEBCO bathymetry.
351 Fig 6. Post-nesting (2014) and remigration (2016) of hawksbill turtle 137659 from nesting beach,
352 Tortuguero, Costa Rica, to foraging habitat in northern Nicaragua. Map was made in ArcGIS Pro
353 using GADM shapefiles (https://gadm.org/index.htm) and GEBCO bathymetry.
354 From GMNWR, turtle 107904 traveled 970 km in 26 days ending at Half Moon Reef (Table 2;
355 Fig 7). Turtle 107915 traveled from GMNWR to Utila, Honduras to forage between Utila and the
356 mainland, a migration that took 44 days and covered 1,522 km (Fig 7). We tracked turtle 107910
357 for 605 km over 14 days, where she reached a foraging habitat between Corn Island and San
358 Andreas/San Luis, in central Nicaragua (Fig 7). Finally, turtle 107913 looped out away from the
359 shore into the Colombia-Panama Gyre before returning to move along the coastline in central
360 Costa Rica and continuing north to Half Moon Reef, a trip that took 46 days and covered 1,494
361 km (Table 2, Fig 7).
362 Fig 7. Migrations of hawksbill sea turtles nesting on Gandoca-Manzanillo National Wildlife
363 Refuge, Costa Rica, in 2018, and one turtle nesting on Chiriqui Beach, Panama in 2017. Map
364 was made in ArcGIS Pro using GADM shapefiles (https://gadm.org/index.htm) and GEBCO
365 bathymetry.
366 The single turtle that nested on CBP (160923) traveled 924 km in 30 days to forage alongside the
367 Costa Rican hawksbills on the Nicaraguan Rise (Table 2; Fig 7).

## 368 Foraging habitat ARS

369 The longest recorded foraging period was 1,198 days, and foraging time seemed to be habitat
370 specific, with turtle 137659 returning to the nesting beach (remigration) after less than two years
371 in her foraging habitat (598 d), and turtle 107915 remaining in her foraging habitat for more than
372 1,198 days. Turtle 107915 was recorded foraging in a primary foraging area for 784 days before

moving to a second foraging area for 414 days until the transmitter failed.

375 Hawksbill turtles nesting at PHB used coastal foraging habitats within Honduras, Belize, and
376 Mexico in areas that included reefs, shallow water, and sometimes coastal inlets (Fig 8). Core
377 foraging ARS ranged from 6 km2 to 441 km2 (Table 2) and were generally smaller than that of
378 inter-nesting core ARS (Table 1). Turtle 120378 engaged in coastal wandering which we
379 included in the UD calculations, resulting in a foraging core use area larger than the other
380 foraging UDs. We did not have long-term recording of foraging location data from the PHB
381 turtles (< 63 d). Taken together, core foraging area for turtles nesting at PHB was 99 ± 192 km^2^.
382 Fig 8. Foraging area restricted search (95% UD with 50% UD bolded inset) behavior of
383 hawksbill sea turtles nesting on Pumpkin Hill Beach, Utila, Honduras (pink); Tortuguero, Costa
384 Rica (orange); Gandoca-Manzanillo National Wildlife Refuge, Costa Rica (red); and Chiriqui
385 Beach, Panama (brown). Map was made in ArcGIS Pro using GADM shapefiles
386 (https://gadm.org/index.htm) and GEBCO bathymetry.
387 Turtles tagged at TNP foraged along the Nicaraguan continental shelf (Fig 8). These turtles had
388 core foraging UD that ranged from 14 km2 and 705 km2 (Table 2). Each turtle had a smaller
389 foraging UD compared to their respective inter-nesting UD. Turtle 137659 had the longest
390 recorded number of foraging days (598 d) before migrating back to TNP to nest again. The core
391 foraging area for this turtle (14 km^2^) was the smallest used by any of the turtles tracked from
392 TNP. On average, core foraging area was 227 ± 273 km^2^.
393 Turtles nesting at GMNWR foraged in the same habitat as the turtles from TNP (n = 3) and
394 adjacent to PHB (n = 1; Fig 8). Turtles that foraged along the continental shelf of Nicaragua were
395 tracked between 263 and 463 days (Table 1) and had core UDs of between 23 km2 and 260 km^2^
396 (Table 2). Turtle 107915 foraged south of Utila Island (Fig 8). For the first 784 days the turtle
397 had a primary foraging core UD of 23 km^2^, then moved west approximately 100 km and spent
398 414 days in a secondary foraging UD of 27 km2 (Table 2). This turtle did not return to the initial
399 area for the duration of signal transmissions. Overall, mean foraging area for turtles nesting at
400 GMNWR was 84 ± 106 km^2^.
401 Finally, the turtle tagged on CBP also foraged in the same habitat as turtles from TNP and
402 GMNWR, along the Nicaragua Rise (Fig 8). This turtle was tracked for 472 days and had a very
403 small core UD of 6 km2 (Table 2).

## 404 Influence of currents on turtle movements

405 Cosine similarity (*SC*) measures the angle of the direction of the turtle's movement and the ocean
406 current movement. Of the turtles nesting on PHB, turtle 108759 traveled against ocean currents
407 between the island and the mainland of Belize, and then with ocean currents up the coast to
408 Mexico. This led to an average weighted (by track segment length) *SC* between the track
409 direction and the ocean current direction of 0.07 (Table 2, Fig 9). The with-current movement
410 and against-current movement canceled each other out. The second turtle (108760) traveled
411 predominantly against prevailing ocean currents (weighted *SC* of −0.33) to Middle Long Caye,
412 Belize (Table 2). Turtle 119995 traveled perpendicular to ocean current movement and stopped
413 off the west coast of Utila (weighted *SC* = 0.003; Table 2). Turtle 120378 zigzagged across ocean
414 currents to Laughing Bird Caye in a way that averaged overall weighted *SC* (−0.01; Table 2).
415 Finally, turtle 138816 traveled mostly perpendicular to ocean currents the entire time to the

Drowned Cayes (weighted *SC* = 0.04; Table 2).

418 Fig 9. Daily cosine similarity between turtle migration direction and ocean current direction for
419 turtles tagged on Pumpkin Hill Beach, Utila, Honduras. Cosine similarity close to +1 indicates
420 that the direction of turtle movement was with ocean currents, whereas a cosine similarity close
421 to −1 indicates a turtle movement direction against ocean currents. In the figure, sections in
422 green were designated as generally with the currents, sections in yellow were designated as
423 generally across the currents, and sections in red were generally against ocean currents. Each
424 panel represents the migration of a single turtle whose satellite tag number is provided on the left
425 above each panel.
426 From TNP, turtle 22126’s migration direction had a weighted *SC* = 0.06 compared to ocean
427 current direction ending in central Nicaragua, while turtle 22134 had a weighted *SC* = 0.09 and
428 ended in northern Nicaragua; both traveled mostly against the water current (Fig 10). Turtle
429 137659 migrated to the continental shelf off Northern Nicaragua, then remigrated two years later
430 to nest in TNP. The post-nesting migration swapped back and forth across the current, so that the
431 turtle spent a few days in each direction (weighted *SC* = −0.02; Table 2). The remigration,
432 however, was 8 days against the current with one day (day 6) traveling with the current until the
433 turtle was able to catch a current and spend the last 7 days traveling with the currents (weighted
434 *SC* = −0.21; Fig 10). Turtle 129347 migrated with water current direction for 12 out of 18 days
435 (weighted *SC* = 0.05; Fig 10) to reach the continental shelf of central Nicaragua. Finally, turtle
436 220791 travelled across ocean currents (weighted *SC* = 0.04) to forage on Half Moon Reef, along
437 the Nicaragua Rise on the continental shelf adjacent to the border of Nicaragua and Honduras.
438 Fig 10. Daily cosine similarity between turtle migration direction and ocean current direction for
439 turtles tagged on Tortuguero, Costa Rica. Cosine similarity close to +1 indicates that the
440 direction of turtle movement was with ocean currents, whereas a cosine similarity close to −1
441 indicates a turtle movement direction against ocean currents. In the figure, sections in green were
442 designated as generally with the currents, sections in yellow were designated as generally across
443 the currents, and sections in red were generally against ocean currents. Each panel represents the
444 migration of a single turtle whose satellite tag number is provided on the left above each panel.
445 For turtle 137659, both a post-nesting migration and a remigration are presented.
446 At GMNWR, all of the turtles seemed to ride the Colombia-Panama Gyre out and then returned
447 to the coast before turning north. Turtles 107904 and 107910 returned to the continental shelf in
448 southern Nicaragua and then traveled north, ending at the Nicaraguan Rise (weighted *SC* = 0.08)
449 and central Nicaragua continental shelf (weighted *SC* = −0.10), respectively, a journey that was
450 mostly with the direction of the currents, although ending in central Nicaragua required a final
451 against-current movement (Fig 11). Turtle 107915 also returned near shore around central
452 Nicaragua, but continued around the horn of Nicaragua to Honduras, a journey that followed the
453 direction of the local ocean currents (weighted *SC* = 0.09). Finally, turtle 107913 returned to the
454 shore in central Costa Rica, requiring more days swimming against ocean currents before turning
455 to move with the currents along the coast to the Nicaragua Rise and Half Moon Reef (weighted

456	*SC* = 0.05).

457 Fig 11. Daily cosine similarity between turtle migration direction and ocean current direction for
458 turtles tagged on Gandoca-Manzanillo National Wildlife Refuge, Costa Rica, and Chiriqui
459 Beach, Panama (160923). Cosine similarity close to +1 indicates that the direction of turtle
460 movement was with ocean currents, whereas a cosine similarity close to −1 indicates a turtle
461 movement direction against ocean currents. In the figure, sections in green were designated as
462 generally with the currents, sections in yellow were designated as generally across the currents,
463 and sections in red were generally against ocean currents. Each panel represents the migration of
464 a single turtle whose satellite tag number is provided on the left above each panel.
465 From CBP, turtle 160923 traveled to the Nicaragua Rise and had an average weighted *SC* = 0.08,
466 mostly travelling perpendicular and against the currents (Fig 11).

# 467 Discussion

468 Although prior reports by Godley et al. (21) and Hays and Hawkes (23) recognized that most
469 satellite telemetry had been accomplished on loggerheads (*C. caretta*), greens (*C. mydas*), and
470 leatherbacks (*D. coriacea*), recent studies by Maurer et al. (32), and a compilation of some 258
471 tracks from 16 countries and overseas territories by Maurer et al. (30), have vastly increased our
472 understanding of inter-nesting and post-nesting movements of hawksbills in the Western Atlantic
473 over the last 10 years. Nevertheless, tracking data from the Central American countries of Belize,
474 Honduras, Nicaragua, and Costa Rica are generally lacking in assessments of telemetry studies.
475 Here we present results that begin to fill gaps in our knowledge for hawksbills

nesting in Honduras, Costa Rica, and Panama.

478 We used satellite telemetry to track hawksbill movements from four nesting beaches in three
479 countries along the far western region of the Caribbean at different times between 2000 – 2022,
480 in which IN area use was generally close to the nesting beaches and was larger than foraging
481 area size. Migration paths zig-zagged across currents, possibly in order to limit against-current
482 movement. Foraging areas were located along the continental shelves of Nicaragua, Honduras,
483 Belize, and Mexico.

## 484 Inter-nesting

485 In this study, we observed a wide range of IN area use, from a core area use of 4 km2 over a
486 period of 65 days near CBP, to 2,643 km2 over a period of 21 days off the coast of TNP. The
487 continental shelf in Costa Rica is narrow, and area use indicates that turtles spent time offshore
488 in deep water, adjacent to the Colombia-Panama Gyre. This current system may prevent the
489 turtles from resting in smaller areas as there is less geographic protection compared to Honduras,
490 which has shallow waters < 100 m deep, or Panama, where the continental shelf is shallow and
491 allowed the entire residential UD to be well within the 50 m depth contour. Small IN area use
492 estimated for PHB and CBP turtles are common for hawksbill turtles in Caribbean islands, such
493 as the Dominican Republic (33), St. Croix (35), Jamaica, and Antigua (32). The IN area use for
494 turtles at TNP were larger than those found at the other sites, suggesting that currents may pull
495 turtles away from the nesting beach, creating an environment that potentially lacks sufficient
496 resting areas resulting in more active IN periods. This is likely to have significant energetic costs
497 for turtles, especially if they are fasting during the nesting season. For example, turtles nesting at
498 GMNWR were all swept out into the Colombia-Panama Gyre upon beginning their migrations. If
499 this were to happen to a turtle during IN attempts, it may potentially be prohibitive to subsequent
500 nesting efforts.

## 501 Post-nesting migrations

502 Post-nesting hawksbills in previous studies from the Caribbean basin have demonstrated two
503 main strategies of migration back to foraging sites, with some migrating internationally to arrive
504 at foraging sites long distances (respectively defined according to the study) from the nesting
505 grounds (14, 19, 31-35, 59-61), while others remained nationally in areas proximal to their
506 nesting grounds (14, 19, 31-33, 60). Interestingly, the majority of turtles migrating from the
507 insular Caribbean head west, southwest, or less frequently northwest (although see van Dam et

al. (19), Moncada et al. (31), and Hart et al. (35)).

510 All turtles in our study undertook regional, although relatively short-distance migrations, and all
511 utilized migration corridors along the far Western Caribbean, making use of the relatively
512 shallow continental shelf zones from Panama up to the peninsula of Mexico. Even regional,
513 relatively short-distance migrations require large amounts of metabolic energy, potentially
514 impacting reproductive outputs and reducing the amount of foraging time available upon
515 reaching the foraging site. However, there may be advantages (and presumably selective
516 pressures) to maintaining fidelity to distant foraging grounds. Shimada et al. (62) suggest prior
517 knowledge of the foraging area and an ability to survive long-term are definite benefits, likely to
518 outweigh haphazardly establishing new foraging sites that may indeed have adequate food
519 availability, yet whose long-term survivability is unknown. Additionally, the unknown factor of
520 encountering predators in a new foraging habitat may reduce the suitability of the habitat (63),
521 especially for turtles that have already experienced years of site suitability at their existing
522 foraging grounds.
523 Coastal migrations are not always the most advantageous choice for migrating when water
524 currents are strong. In our study, turtles nesting south of central Costa Rica instead chose longer
525 routes that took advantage of circular current movements. This involved either being swept out
526 by the Colombia-Panama Gyre, or actively swimming with the currents of the Gyre, then
527 diverting away from it towards the coast, terminating somewhere along the Nicaraguan or north
528 Costa Rican coasts. This route may mimic the path turtles took as hatchlings when they dispersed
529 from their natal beaches for the first time and were swept out into ocean currents (64). This idea
530 may shed light on how they initially found their adult foraging habitats, since there is ample
531 experimental and observational evidence demonstrating the ability of sea turtles to detect and
532 orient to magnetic fields throughout their pelagic (65-67), neritic (68, 69), and adult (70, 71)
533 stages. Nevertheless, more research on this aspect of foraging-ground finding is needed. In any
534 case, adult turtles exited the Gyre at several different places, with some turtles returning to the
535 shoreline in Nicaragua and swimming north, while another turtle (107913) remained in the Gyre
536 back to central Costa Rica before turning north. Eventually, these turtles moved into foraging
537 areas along Nicaragua and Honduras, most notably the Nicaragua Rise.
538 Our findings support Maurer and Eckert (30) that there is mounting evidence that the region off
539 the northeast coast of Honduras along the Nicaragua Rise may well be a regional foraging
540 “hotspot.” This area has been highlighted in prior studies as an area that is important not only for
541 post-nesting hawksbill turtles, but also green (*C. mydas*) (72) and loggerhead (*C. caretta*) (73)
542 turtles as foraging grounds (74). Further, this foraging area is important for hawksbills from
543 throughout the Caribbean (14, 18, 31, 33-35). Becking et al. (61) found post-nesting turtles from
544 Bonaire traveled as much as 1,766 km (*C. caretta*) and 3,135 km (*E. imbricata*) to reach this
545 foraging area, while Maurer et al. (32) found post-nesting hawksbills from Jamaica traveled
546 more than 723 km to reach and settle in the Nicaragua Rise area off the coast of Honduras. Given
547 the paucity of ecological information available for this location, evidence from migrating nesting
548 turtles in our study support the need for intensive investigations into habitat features, fishing
549 pressures, resident population dynamics, and movement strategies of turtles in this potentially
550 important foraging residency area.
551 One hawksbill from Costa Rica (107915) continued its migration along the continental shelf of
552 Honduras to an area of open water approximately 50 km southeast of Utila where it remained for
553 more than 700 days before moving west to an area just 3.5 km off the coast of Punta Izopo
554 National Park, where it was tracked for an additional 400 days. This behavior is similar to that
555 reported by Maurer et al. (32) of a post-nesting hawksbill from Jamaica undertaking migrations
556 to two separate locations, remaining in the first for approximately 71 days before moving on to
557 the second 403 km further south. In contrast, in our study turtle 107915 showed extremely long
558 residence times in both locations. We are unsure why this turtle took up such long residence
559 times in two different locations only approximately 52 km apart, and there is little available
560 information regarding the health and suitability of reef habitats and abundances of prey species
561 for hawksbills in these locations. However, the second site where this turtle remained is within
562 the marine area of the Jeannette Kawas National Park (JKNP), and therefore may provide some
563 level of protection to turtles that forage there, although only recommendations have been
564 proposed to fully protect the area of the park (75) and, to our knowledge, no legislation to protect
565 and patrol the park since that time has been forthcoming. If the area of the JKNP has not moved
566 beyond the status of a ‘paper park,’ turtles who forage in the area may be threatened by illegal
567 direct take, as they are in other areas of coastal Caribbean Honduras.
568 It is of interest that none of the turtles tracked from PHB navigated southeast to forage in the
569 same area as other adults foraging in the Cordelia Banks/Cayos Cochinos area, or northeast in
570 the Nicaragua Rise hotspot. Instead, all of them moved west or northwest along the continental
571 shelf, hugging the coastal zone. The entire coastline from the Yucatan through the Gulf of
572 Honduras appears to have reserve areas important as foraging grounds for migrating hawksbills
573 from coastal Central America, as well as the insular Caribbean (30, 32, 61), demonstrating the
574 necessity of protected zones that may help mitigate the effects of legal and illegal take, inshore
575 fisheries, and large-scale fisheries bycatch.

## 576 Foraging home ranges

577 We found hawksbills making post-nesting migrations to foraging grounds had very small
578 foraging habitats. These data infer that small area use may indicate high-quality habitats or food
579 resources, sufficient resting sites, and stable temperatures that do not require turtles to move to
580 thermoregulate. We tracked turtles within foraging habitats over multiple years; therefore, our
581 results suggest they do not need to seek out new foraging grounds in order to acquire sufficient
582 food resources. With the exception of one turtle from Costa Rica (107915) who established two
583 different foraging home ranges with core UDs of 23 km2 and 27 km^2^, respectively (possibly due
584 to insufficient prey abundances), the foraging home ranges turtles settled into appear to contain
585 sufficient foraging resources. For Turtle 107915, we presume the primary foraging area had
586 insufficient prey abundances to meet the energy needs of a single nesting female over the long
587 term. Just to the north of this area, studies by Baumbach et al. (76) and Wright et al. (77) have
588 demonstrated juvenile populations of hawksbills have small home ranges (<1 km^2^) and suggest
589 these small home ranges are sufficient for juvenile growth because of the high relative
590 abundances of food species, including sponges (especially *Geodia neptuni*) and the red algae
591 *Kalymenia leminguii*. For the other turtles we tracked, our core UD foraging home range data
592 support the findings of other studies that have demonstrated small foraging home ranges of 2 –

593	50 km2 (59, 78, 79).

594

595 The foraging UDs we report in the current study are different than some hawksbills tracked from
596 the Dominican Republic by Hawkes et al. (14), who found turtles settled in foraging home
597 ranges (using α-hulls) of from >1,000 – 4,422 km^2^, in deep waters (>500 m) located from 4 –
598 195 km off the coast. Although some turtles in the current study migrated to habitats ~ 180 km
599 off the coast (i.e. the Nicaragua Rise), they nevertheless maintained home ranges a fraction of the
600 size of those for turtles from the Dominican Republic.

## 601 Currents

602 Few studies have investigated the direction of travel of hawksbill turtles during post-nesting
603 migrations in relation to surface currents. In the current study, we report detailed swim direction
604 of hawksbills as daily *SC* plots for turtles and current directions. Our results demonstrate that
605 most turtles from Honduras and Tortuguero, Costa Rica began their migrations either cutting
606 across prevailing currents, or swimming directly against them. This differs from most turtles
607 tracked from GMNWR, Costa Rica, and Panama, who began their migrations by following the
608 currents out into the ocean, then shifting between moving with and moving against currents
609 multiple times through the remainder of their migrations. Additionally, turtles from the two more
610 northern nesting grounds appeared to have fewer large changes in *SC,* representing moving with
611 versus moving against currents, than did turtles from the more southern nesting grounds. This,
612 however, may be a function of the much longer migration lengths for turtles from both GMNWR
613 and Panama compared with those from Honduras and TNP. The movements of turtle 107910
614 from GMNWR, whose shorter tracking duration was similar to those from the northern beaches,
615 supports this hypothesis, as she demonstrated fewer shifts between moving with verses against

the current.

618 Despite differences in nesting beaches, foraging habitats, and year of migration, all hawksbill
619 turtles that we tracked displayed post-nesting migration directions that averaged close to a
620 weighted *SC* of zero. Each turtle accomplished this via a different movement strategy, whether it
621 involved traveling across currents for the entire migration, or dramatic zigzagging that changed
622 relative direction every day (Figs 9, 10, 11). The largest variations to this pattern were turtles
623 108759 and 108760 that seem to have only swam with ocean currents. Another point of interest
624 was that the re-migration of turtle 137659 involved traveling back to Tortuguero from her
625 foraging habitat. In this case, the re-migration corridor was almost the post-nesting corridor in
626 reverse; however, the weighted *SC* was much closer to zero than expected. We are unable,
627 therefore, to assume that this turtle traveled with currents in one direction and against currents in
628 the other direction. Instead, she was able to energetically mediate her efforts in regard to ocean
629 movements in both the post-nesting migration and re-migration back to the nesting beach.
630 Our interpretations of migratory movements in relation to currents agrees well with reports by
631 Horrocks et al. (59) of post-nesting hawksbills leaving Barbados cutting across southeasterly
632 currents to avoid running headlong into northwesterly currents as the turtle was migrating
633 northwest to Dominica. Additionally, those authors report another turtle moving southwest from
634 Barbados cutting across both southeastern and northeastern currents, and ranging far west of the
635 target foraging grounds in Trinidad, presumably allowing the turtle to avoid strong counter-
636 currents for much of the migration south (59). We agree that although turtles are fully capable of
637 swimming against prevailing currents and tides over long distances (80), cross-cutting and
638 utilizing current flows in the direction of migration are likely to reduce demands on depleted
639 energy stores following months of mating and nesting activities. Taking advantage of prevailing
640 currents may serve to minimize energy expenditure and travel times required to reach foraging
641 sites (59). This is further supported through work by Mencacci et al. (81), who tracked post-
642 nesting female loggerhead turtles (*C. caretta*) displaced for the purpose of the study, in relation
643 to oceanic sea surface temperature (SST) and geostrophic velocities. Those authors found turtles
644 generally moved in the direction of prevailing currents, with few instances in which turtles
645 clearly moved against currents, and where migratory routes were heavily influenced by oceanic
646 features turtles encountered. Further, they found turtles appeared to actively swim in the same
647 direction of currents, rather than passively drift along with prevailing currents (81). Actively
648 swimming in the same direction as currents, even during intermittent periods of long-distance
649 migrations, is likely to increase swimming rates and reduce the overall duration of migrations
650 between nesting and foraging grounds. This may explain some of what Hays et al. (82) found
651 when they recorded turtles migrating towards islands based on course headings. While their
652 subsequent model suggested that turtles were not being carried off course by currents, it would
653 be interesting to test whether energetically canceling out ocean current movement is involved in
654 the apparent course navigational system of sea turtles.

## 655 Conservation implications

656 The importance of enacting multi-national agreements to protect sea turtles from direct take as
657 well as fisheries bycatch along their migration routes is supported by our data, adding to the data
658 collected in the greater Caribbean region (14, 19, 30-35, 59-61). Our study further stresses that
659 foraging hotspots, such as those in the Nicaragua Rise, the Gorda Banks off Honduras’ northeast
660 coast, and the Cayos Miskitos off northern Nicaragua, require further investigation to better
661 understand the importance of these habitats as foraging grounds for migrating turtles. Ongoing
662 threats, such as direct legal take (83, 84), and large- and small-scale fisheries bycatch (83-85)
663 exist for the turtles in this study, who maintain migration routes along the shallow Inter-
664 American coastal shelf. While these threats may, to some degree, be mitigated by the presence of
665 established marine protected areas along the Central American coastline and Meso-American
666 Barrier Reef, a large percentage of these are poorly funded, are undermanaged, and have little
667 evidence demonstrating their effectiveness in protecting endangered marine species (33, 86-88).
668 The efficacy of the protection provided to turtles by MPAs will depend on international
669 cooperation and intentional effort to mitigate the effects of both legal and illegal fisheries found
670 in sea turtle migration corridors. These efforts, in turn, will need to be supported by data
671 elucidating the small-scale, high-resolution movement of turtles within and outside of these
672 protected zones. We suggest there is a need for continued satellite tracking of nesting hawksbill
673 turtles from locations throughout Central America and the insular Caribbean with higher
674 resolution/accuracy locations to fill the gaps in our knowledge regarding hawksbill nesting
675 beaches, migration corridors, and foraging grounds in the Western Atlantic.

# 677 Acknowledgements

678 Funding for this project was provided by the Boyd Lyon Sea Turtle Fund, the Sonoma County
679 Community Foundation, the Fort Wayne Children's Zoo Conservation Fund and the Jack Schrey
680 Distinguished Professor Funds, the USFWS Marine Turtle Conservation Fund under the
681 direction of Earl Possardt, the Department of Earth and Biological Sciences, Loma Linda
682 University, as well as the California Turtle and Tortoise Club Inland Empire Chapter. We would
683 also like to extend special thanks to ProTECTOR, Inc. Country Director, Lidia Salinas for
684 securing research permits #s DGPA-005-2006 and DGPA-245-2006 in Honduras, Jimmy Miller
685 for logistical support on Roatan, and Chase Kent-Dotson for his assistance with data analyses.
686 This Contribution No.	of the Marine Research Group (LLU) and Contribution No.	of

ProTECTOR, Inc.

# 697 References

698 1.	Witzell WN. Synopsis of biological data on the hawksbill turtle *Eretmochelys imbricata*
699 Linnaeus, 1766. Food and Agriculture Organization Fisheries Synopsis No 137. 1983:1.
700 2.	Parsons JJ. The hawksbill turtle and the tortoise shell trade. Études de Géographie Tropicale
701 Offertes à Pierre Gourou. Paris: Mouton; 1972. p. 45 - 60.
702 3.	Pritchard PCH. Evolution, Phylogeny, and Current Status. In: Lutz PL, Musick JA, editors. The
703 Biology of Sea Turtles. I. Boca Raton, FL: CRC Press; 1997. p. 1 - 28.
704 4.	Fleming EH. Swimming Against the Tide: Recent Surveys of Exploitation, Trade, and
705 Management of Marine Turtles in the Northern Caribbean. Washington, D.C., USA: TRAFFIC
706 North America; 2001. 185 p.
707 5.	Mortimer JA, Donnelly M. IUCN Red List Status Assessment, Hawksbill Turtle (*Eretmochelys*
708 *imbricata*). Washington, DC.: IUCN/SSC Marine Turtle Specialist Group,; 2007.
709 6.	Meylan AB, Donnelly M. Status justification for listing the Hawksbill Turtle (*Eretmochelys*
710 *imbricata*) as critically endangered on the 1996 IUCN Red List of Threatened Animals.
711 Chelonian Conservation and Biology. 1999;3(2):200 - 24.
712 7.	IUCN. IUCN Red List 2024 [Available from:
713 http[s://www.iucnredlist.org/specie](http://www.iucnredlist.org/species/8005/12881238)s/80[05/12881238.](http://www.iucnredlist.org/species/8005/12881238)]
714 8.	Hays GC, Broderick AC, Godley BJ, Luschi P, Nichols WJ. Satellite telemetry suggests high
715 levels of fishing-induced mortality in marine turtles. Marine Ecology Progress Series. 2003;

716	262:305 - 9.

717 9.	Dutton PH, Squires D. Reconciling biodiversity with fishing: a holistic strategy for Pacific sea
718 turtle recovery. Ocean Development and International Law. 2008;39(2):200 - 22.
719 10.	Wallace BP, DiMatteo AD, Bolten AB, Chaloupka MY, Hutchinson BJ, Abreu-Grobois FA, et al.
720 Global Conservation Priorities for Marine Turtles. PLoS ONE. 2011;6(9):e24510.
721 11.	The Laúd OPO Network. Enhanced, coordinated conservation efforts required to avoid
722 extinctionof critically endangered Eastern Pacific leatherback turtles. 2020. Contract No.:

723	1(2020).

724 12.	Pendoley KL, Schofield G, Whittock PA, Ierodiaconou D, Hays GC. Protected species use of a
725 coastal marine migratory corridor connecting marine protected areas. Marine Biology. 2014.
726 13.	Marcovaldi MA, Lopez GG, Soares LS, Lima EHSM, Thome JCA, Almeida AP. Satellite-
727 tracking of female loggerhead turtles highlights fidelity behavior in northeastern Brazil.
728 Endangered Species Research. 2010;12:263 - 72.
729 14.	Hawkes LA, Tomas J, Revuelta O, Leon YM, J. M. Blumenthal JM, Broderick AC, et al.
730 Migratory patterns in hawksbill turtles described by satellite tracking. Marine Ecology-Progress

731	Series. 2012;461:223 - 32.

732 15.	Carr AF. Handbook of Turtles; The Turtles of the United States, Canada, and Baja California.
733 Ithaca, New York: Cornell University Press; 1952. 557 p.
734 16.	Plotkin P. Adult Migrations and Habitat Use. In: Lutz PL, Musick JA, Wyneken J, editors. The
735 Biology of Sea Turtles Volume II. Boca Raton, FL: CRC Press; 2003. p. 225 - 41.
736 17.	Meylan AB. International movements of immature and adult hawksbill turtles (*Eretmochelys*
737 *imbricata*) in the Caribbean Region. Chelonian Conservation and Biology. 1999;3(2):189 - 94.
738 18.	Troëng S, Dutton PH, Evans DR. Migration of hawksbill turtles *Eretmochelys imbricata* from
739 Tortuguero, Costa Rica. Ecography. 2005;28:394 - 402.
740 19.	van Dam RP, Diez CE, Balazs GH, Colon Colon LA, McMillan WO, Schroeder B. Sex-specific
741 migration patterns of hawksbill turtles breeding at Mona Island, Puerto Rico. Endangered Species

742	Research. 2008;4:85 - 94.

743	20.	Espinosa CO, Meylan AB, Meylan PA, Peterson I, Diez CE, van Dam RP. Hawksbill tagged as a

744	juvenile in Puerto Rico found nesting in Panama 15 years later. Marine Turtle Newsletter.

745	2010;127 (april 2010):25.

746	21.	Godley BJ, Blumenthal JM, Broderick AC, Coyne MS, Godfrey MH, Hawkes LA, et al. Satellite

747	tracking of sea turtles: Where have we been and where do we go next? Endangered Species

748	Research. 2008;4:3 - 22.

749 22.	Hart KM, Hyrenbach KD. Satellite telemetry of marine megavertebrates: the coming of age of an
750 experimental science. . Endangered Species Research 2009;10:9 - 20.
751 23.	Hays GC, Hawkes LA. Satellite tracking sea turtles: opportunities and challenges to address key
752 questions. Frontiers in Marine Sciences. 2018;5:432.
753 24.	Dampier W, Gray A. A New Voyage Around the World. Dover Edition ed. New York: Dover
754 Publications; 1968.
755 25.	Bjorndal KA, Archie Carr A, Meylan AB, Mortimer JA. Reproductive biology of the hawksbill
756 *Eretmochelys imbricata* at Tortuguero, Costa Rica, with notes on the ecology of the species in the
757 Caribbean. Biological Conservation. 1985;34:353 - 68.
758 26.	Meylan AB. Status of the Hawksbill Turtle (*Eretmochelys imbricata*) in the Caribbean Region.
759 Chelonian Conservation and Biology. 1999;3(2):177 - 84.
760 27.	McClenachan L, Jackson JBC, Newman MJH. Conservation implications of historic sea turtle
761 nesting beach loss. Frontiers in Ecology and the Environment. 2006;4(6):290 - 6.
762 28.	Alvarado JJ, Cortés J, Esquivel MF, Salas E. Costa Rica’s Marine Protected Areas: status and
763 perspectives. Revista de Biología Tropical. 2012;60(1):129 - 42.
764 29.	Figgener C. Project for the Conservation of sea turtles, Playa Gandoca Hawksbill Turtle and
765 Green Turtle Season. Costa Rica: WIDECAST; 2009.
766 30.	Maurer AS, Eckert KL. Distributions of the Hawksbill Sea Turtle (*Eretmochelys imbricata*) in the
767 Western Atlantic Inferred from Satellite Telemetry. Godfrey, Illinois: UNEP Caribbean
768 Environment Programme, Specially Protected Areas and Wildlife Regional Activity
769 Centre (SPAW RAC); 2024.
770 31.	Moncada FG, Hawkes LA, Fish MR, Godley BJ, Manolis SC, Medina Y, et al. Patterns of
771 dispersal of hawksbill turtles from the Cuban shelf inform scale of conservation and management.
772 Biological Conservation. 2012;148(1):191-9.
773 32.	Maurer AS, Dawson C, Bjorkland R, Donaldson A, Stapleton SP, Richardson JI, et al. Satellite
774 Telemetry Elucidates Migratory Pathways and Foraging Areas for Hawksbill Sea Turtles,
775 *Eretmochelys imbricata*, in the Caribbean. Caribbean Journal of Science. 2022; 52(1):126-41, 16.
776 33.	Revuelta O, Hawkes L, León YM, Godley BJ, Raga JA, Tomás J. Evaluating the importance of
777 Marine Protected Areas for the conservation of hawksbill turtles *Eretmochelys imbricata* nesting
778 in the Dominican Republic. Endangered Species Research. 2015;27(2):169-80.
779 34.	Nivière M, Chambault P, Pérez T, Etienne D, Bonola M, Martin J, et al. Identification of marine
780 key areas across the Caribbean to ensure the conservation of the critically endangered hawksbill
781 turtle. Biological Conservation. 2018;223:170-80.
782 35.	Hart KM, Iverson AR, Benscoter AM, Fujisaki I, Cherkiss MS, Pollock C, et al. Satellite tracking
783 of hawksbill turtles nesting at Buck Island Reef National Monument, US Virgin Islands: Inter-
784 nesting and foraging period movements and migrations. Biological Conservation. 2019;229:1-13.
785 36.	Meylan A. Spongivory in Hawksbill Turtles - a Diet of Glass. Science. 1988;239(4838):393-5.
786 37.	Berube MD. Home Range and Foraging Ecology of Juvenile Hawksbill Sea Turtles Around
787 Roatan, Honduras: Loma Linda University; 2010.
788 38.	Baumbach DS, Zhang R, Hayes CT, Wright MK, Dunbar SG. Strategic foraging: Understanding
789 hawksbill (*Eretmochelys imbricata*) prey item energy values and distribution within a marine
790 protected area. Marine Ecology. 2022;43(2):e12703.
791 39.	Carrión-Cortez J, Canales-Cerro C, Arauz R, Riosmena-Rodríguez R. Habitat use and diet of
792 juvenile eastern Pacific hawksbill turtles (*Eretmochelys imbricata*) in the North Pacific coast of
793 Costa Rica. Chelonian Conservation and Biology. 2013;12(2):235 - 45.
794 40.	Dunbar SG, Randazzo A, Salinas L, Luque J. Final Report of the Protective Turtle Ecology
795 Center for Training, Outreach, and Research, Inc. on Community-Directed Capacity Building for
796 Hawksbill Conservation and Population Recovery in Caribbean Honduras. Loma Linda, CA:
797 United States Fish and Wildlife Service - Marine Turtle Conservation Fund (USFWS-MTCF);

798	2013.

799 41.	Damazo LRE. Nesting Ecology of Hawksbill Sea Turtles (*Eretmochelys imbricata*) on Utila,
800 Honduras. Loma Linda, CA. USA: Loma Linda University; 2014.
801 42.	Stapleton SP, Eckert KL. Community-Based Sea Turtle Research and Conservation in Dominica:
802 A Manual of Recommended Practices. Prepared by the Wider Caribbean Sea Turtle Conservation
803 Network (WIDECAST) and the Dominica Sea Turtle Conservation Organization (DomSeTCO),
804 with funding from the United States Agency for International Development. Beaufort, North
805 Carolina: WIDECAST; 2008. Report No.: WIDECAST Technical Report No. 8.
806 43.	MINAE. Plan de Manejo para el Refugio Nacional de Vida Silvestre Gandoca-Manzanillo. 1996.
807 Contract No.: 07 January 2019.
808 44.	RAMSAR. The Convention on Wetlands 1995 [Costa Rica]. Available from:
809 https://rsis.ramsar.org/ris/783?language=en.
810 45.	Chacón-Chaverri D, Eckert KL. Leatherback sea turtle nesting at Gandoca Beach in Caribbean
811 Costa Rica: management recommendations from fifteen years of conservation. Chelonian
812 Conservation and Biology. 2007;6(1):101 - 10.
813 46.	Dunbar SG, Berube MD. Hawksbill sea turtle nesting beach reconnaissance Roatan, Honduras
814 Final Report. Washington, D.C.: United States Fish and Wildlife Services; 2008 November 28.
815 47.	Balazs GH, Miya RK, Beaver SC, editors. Procedures to attach a satellite transmitter to the
816 carapace of an adult Green turtle. 15th Annual Symposium on Sea Turtle Biology and
817 Conservation; 1996 20 - 25 February; Hilton Head, South Carolina. 1995: NOAA Technical
818 Memorandum.
819 48.	Hart KM, Iverson AR, Benscoter AM, Fujisaki I, Cherkiss MS, Pollock C, et al. Resident areas
820 and migrations of female green turtles nesting at Buck Island Reef National Monument, St. Croix,
821 US Virgin Islands. Endangered Species Research. 2017;32:89 - 101.
822 49.	Wildlife Computers. Attachment Protocol for Kit-000.
823 https://staticwildlifecomputerscom/manuals/Attachment-Kit-000-Turtlepdf. Redmond, WA.
824 USA.
825 50.	Freitas C, Lydersen C, Fedak MA, Kovacs KM. A simple new algorithm to filter marine mammal
826 Argos locations. Marine Mammal Science. 2008;24(2):315 - 25.
827 51.	Luschi P, Hays GC, Del Seppia C, Marsh R, Papi F. The navigational feats of green sea turtles
828 migrating from Ascension Island investigated by satellite telemetry. Proceedings of the Royal
829 Society of London Series B: Biological Sciences. 1998;265(1412):2279 - 84.
830 52.	Hays GC, Luschi P, Papi F, Del Seppia C, Marsh R. Changes in behaviour during the inter-
831 nesting period and post-nesting migration for Ascension Island green turtles. Marine Ecology

832	Progress Series. 1999;189:263 - 73.

833	53.	Parker DM, Balazs GH, King CS, Katahira L, Gilmartin W. Short-range movements of hawksbill

834	turtles (*Eretmochelys imbricata*) from nesting to foraging areas within the Hawaiian islands 1.

835	Pacific Science. 2009;63(3):371 - 82.

836	54.	Jonsen ID, Flemming JM, Myers RA. Robust state-space modeling of animal movement data.

837	Ecology. 2005;86(11):2874 - 80.

838	55.	Jonsen ID, Basson M, Bestley S, Bravington MV, Patterson TA, Pedersen MW, et al. State-space

839	models for biologgers: a methodological road map. Deep Sea Research Part II: Topical Studies in

840	Oceanography. 2013;88:34 - 46.

841 56.	Block BA, Jonsen ID, Jorgensen SJ, Winship AJ, Shaffer SA, Bograd SJ, et al. Tracking apex
842 marine predator movements in a dynamic ocean. Nature. 2011;475(7354):86 - 90.
843 57.	Calenge C. Home range estimation in R: the adehabitatHR package. Saint Benoist, Auffargis,
844 France: Office national de la classe et de la faune sauvage; 2011.
845 58.	Calenge C. The package “adehabitat” for the R software: a tool for the analysis of space and
846 habitat use by animals. Ecological Modelling. 2006;197(3-4):516 - 9.
847 59.	Horrocks JA, Vermeer LA, Krueger B, Coyne M, Schroeder BA, Balazs GH. Migration routes
848 and destination characteristics of post-nesting hawksbill turtles satellite-tracked from Barbados,
849 West Indies. Chelonian Conservation and Biology. 2001;4:107 - 14.
850 60.	Esteban N, van Dam RP, Harrison E, Herrera A, Berkel J. Green and hawksbill turtles in the
851 Lesser Antilles demonstrate behavioural plasticity in inter-nesting behaviour and post-nesting
852 migration. Marine Biology. 2015;162(6):1153-63.
853 61.	Becking LE, Christianen MJA, Nava MI, Miller N, Willis S, van Dam RP. Post-breeding
854 migration routes of marine turtles from Bonaire and Klein Bonaire, Caribbean Netherlands.
855 Endangered Species Research. 2016;30:117-24.
856 62.	Shimada T, Limpus C, Jones R, Hazel J, Groom R, Hamann M. Sea turtles return home after
857 intentional displacement from coastal foraging areas. Marine Biology. 2016;163(1):8.
858 63.	Heithaus MR, Frid Ae, Wirsing AJ, Dill LM, Fourqurean JW, Burkholder D, et al. State-
859 dependent risk-taking by green sea turtles mediates top-down effects of tiger shark intimidation in
860 a marine ecosystem. Journal of Animal Ecology. 2007;76(5):837-44.
861 64.	Hays GC, Fossette S, Katselidis KA, Mariani P, Schofield G. Ontogenetic development of
862 migration: Lagrangian drift trajectories suggest a new paradigm for sea turtles. Journal of The
863 Royal Society Interface. 2010;7(50):1319 - 27.
864 65.	Lohmann KJ, Putman NF, Lohmann CMF. The magnetic map of hatchling loggerhead sea turtles.
865 Current Opinion in Neurobiology. 2012;22:336 - 42.
866 66.	Lohmann KJ, Johnsen S. The neurobiology of magnetoreception in vertebrate animals. Trends in

867	Neurosciences. 2000; 23(4):153-9.

868 67.	Putman Nathan F, Endres Courtney S, Lohmann Catherine MF, Lohmann Kenneth J. Longitude
869 Perception and Bicoordinate Magnetic Maps in Sea Turtles. Current Biology. 2011;21(6):463-6.
870 68.	Avens L, Braun-McNeill J, Epperly S, Lohmann KJ. Site fidelity and homing behavior in juvenile
871 loggerhead sea turtles (*Caretta caretta*). Marine Biology. 2003;143(2):211-20.
872 69.	Avens L, Lohmann KJ. Use of multiple orientation cues by juvenile loggerhead sea turtles
873 *Caretta caretta*. Journal of Experimental Biology. 2003;206:4317 - 25.
874 70.	Lohmann KJ, Goforth KM, Mackiewicz AG, Lim DS, Lohmann CMF. Magnetic maps in animal
875 navigation. Journal of Comparative Physiology A. 2022;208(1):41-67.
876 71.	Lohmann KJ, Luschi P, Hays GC. Goal navigation and island-finding in sea turtles. Journal of
877 Experimental Marine Biology and Ecology. 2008;356(1):83-95.
878 72.	Troëng S, Evans DR, Harrison E, Lagueux CJ. Migration of green turtles *Chelonia mydas* from
879 Tortuguero, Costa Rica. Marine Biology. 2005;148::435 - 47.
880 73.	Blumenthal JM, Solomon JL, Bell CD, Austin TJ, Ebanks - Petrie SG, Coyne MS, et al. Satellite
881 tracking highlights the need for international cooperation in marine turtle management.
882 Endangered Species Research. 2006;7:1-11.
883 74.	Ankersen TT, Stocks G, Paniagua F, Grant S. Turtles without borders: The international and
884 domestic law basis for the shared conservation, management, and use of sea turtles in Nicaragua,
885 Costa Rica, and Panama. Journal of International Wildlife Law & Policy. 2015;18(1):1 - 62.
886 75.	Carrasco JC, Secaira E, Lara K. Plan de Conservación del Parque Nacional Blanca Jeannette
887 Kawas Fernández: Basado en Análisis de Amenazas, Situación y del
888 Impacto del Cambio Climático, y Definición de Metas y Estrategias. ICF, USAID ProParque,
889 PROLANSATE, Fundación para la Protección de Punta Sal PIyTP; 2013.
890 76.	Baumbach DS, Anger EC, Collado NA, Dunbar SG. Identifying sea turtle home ranges utilizing
891 citizen-science data from novel web-based and smartphone GIS applications. Chelonian
892 Conservation and Biology. 2019;18(2):133 - 44.
893 77.	Wright MK, Pompe LR, Mishra DR, Baumbach DS, Salinas L, Dunbar SG. Hawksbill presence
894 and habitat suitability of a marine reserve in Honduras. Ocean & Coastal Management.

895	2022;225:106204.

896 78.	Madden Hof CA, Desbiens AA, Kinch J, Fitzsimmons NN, Versace H, Amon A, et al. From
897 rookeries to foraging grounds: understanding regional connectivity and genetic diversity in
898 hawksbill turtles. Frontiers in Marine Science. 2023;10:1201009.
899 79.	Barr CE, Hamann M, Shimada T, Bell I, Limpus CJ, Ferguson J. Post-nesting movements and
900 feeding ground distribution by the hawksbill turtle (*Eretmochelys imbricata*) from rookeries in
901 the Torres Strait. Wildlife Research. 2021;48(7):598 - 608.
902 80.	Luschi P, Hays GC, Papi F. A review of long-distance movements by marine turtles, and the
903 possible role of ocean currents. Oikos. 2003;103(2):293 - 302.
904 81.	Mencacci R, De Bernardi E, Sale A, Lutjeharms JRE, Luschi P. Influence of oceanic factors on
905 long-distance movements of loggerhead sea turtles displaced in the southwest Indian Ocean.

906	Marine Biology. 2010;157(2):339-49.

907	82.	Hays GC, Cerritelli G, Esteban N, Rattray A, Luschi P. Open ocean reorientation and challenges

908	of island finding by sea turtles during long-distance migration. Current Biology.

909	2020;30(16):3236 - 42.

910	83.	Lagueux CJ, Campbell CL, Strindberg S. Artisanal green turtle (*Chelonia mydas*) fishery of

911	Caribbean Nicaragua: II. Characterization and trends in size, sex, and maturity status of turtles

912	killed, 1994–2011. Marine Biology. 2017;164(3):60.

913 84.	Lagueux CJ, Campbell CL, Strindberg S. Artisanal Green Turtle, *Chelonia mydas*, Fishery of
914 Caribbean Nicaragua: I. Catch Rates and Trends, 1991–2011. PLOS ONE. 2014;9(4):e94667.
915 85.	Funes M, Zylich K, Divovich E, Zeller D, Lindop A, Pauly D, et al. Honduras, a fish exporting
916 country: Preliminary reconstructed marine catches in the Caribbean and the Gulf of Fonseca,
917 1950 - 2010. University of British Columbia Vancouver Canada: Fisheries Center; 2015. Report

918	No.: 2015 - 90 Contract No.: 2015-90.

919 86.	Maestro M, Pérez-Cayeiro ML, Morales-Ramírez Á, Chica-Ruiz JA. Evaluation of the
920 management of marine protected areas. Comparative study in Costa Rica. Journal of
921 Environmental Management. 2022;308:114633.
922 87.	Murray R. A governance analysis of three MPAs in Belize: Conservation objectives
923 compromised by tourism development priorities? Marine Policy. 2021;127:104243.
924 88.	Dalton T, Forrester G, Pollnac R. Are Caribbean MPAs making progress toward their goals and

objectives? Marine Policy. 2015;54:69 - 76.

Fig 1	[Click here to access/download;Figure;Fig 1 HiRes Tiff.tif](https://www2.cloud.editorialmanager.com/pone/download.aspx?id=37519517&guid=db00fc87-c3cd-420a-8198-a80e4a6c06b7&scheme=1)

Fig 4	[Click here to access/download;Figure;Fig 4 HiRes Tiff.tif](https://www2.cloud.editorialmanager.com/pone/download.aspx?id=37519534&guid=b6a64ecd-0a39-4a95-b4f2-481948b7d858&scheme=1)

Fig 5	[Click here to access/download;Figure;Fig 5 Hi Res Tiff.tif](https://www2.cloud.editorialmanager.com/pone/download.aspx?id=37519550&guid=9ce7195e-6ac8-4a1e-af88-2e49229e6fcf&scheme=1)

Fig 6	[Click here to access/download;Figure;Fig 6 HiRes Tiff.tif](https://www2.cloud.editorialmanager.com/pone/download.aspx?id=37519552&guid=183b6e4b-3d68-4697-9364-946d10f0a1bf&scheme=1)

Fig 7	[Click here to access/download;Figure;Fig 7 HiRes Tiff.tif](https://www2.cloud.editorialmanager.com/pone/download.aspx?id=37519553&guid=412ddeb8-0a56-4099-a98d-b223f9be469d&scheme=1)

Fig 8	[Click here to access/download;Figure;Fig 8 HiRes Tiff.tif](https://www2.cloud.editorialmanager.com/pone/download.aspx?id=37519555&guid=95287f1f-5532-4356-b3f6-f1dafd14bf1f&scheme=1)

Fig 9	[Click here to access/download;Figure;Fig 9 HiRes Tiff.tif](https://www2.cloud.editorialmanager.com/pone/download.aspx?id=37519564&guid=40dded6a-8d0e-4aaa-8309-49bccf9b6dd3&scheme=1)

Fig 10	[Click here to access/download;Figure;Fig 10 HiRes Tiff.tif](https://www2.cloud.editorialmanager.com/pone/download.aspx?id=37519566&guid=a07e19cf-bb76-4f63-b234-e35e8080d131&scheme=1)

Fig 11	[Click here to access/download;Figure;Fig 11 HiRes Tiff.tif](https://www2.cloud.editorialmanager.com/pone/download.aspx?id=37519567&guid=98f0f122-ec69-444f-b4d3-e88e6593a67b&scheme=1)

-c;;:

107904

0

**0.0**

-05

a

107910,

**0**

.l'I

!!

***U)***

**C**

0-

Migrai1on Cay

107'915

107913

1^1^ 60,9 23

'Migr, ron 1o ay

Fig 2	[Click here to access/download;Figure;Fig 2 HiRes Tiff.tif](https://www2.cloud.editorialmanager.com/pone/download.aspx?id=37533167&guid=8ea7c021-5026-4ba4-a4a1-c79a70c9f4b1&scheme=1)

Fig 3	[Click here to access/download;Figure;Fig 3 HiRes Tiff.tif](https://www2.cloud.editorialmanager.com/pone/download.aspx?id=37533169&guid=b8cf3356-2dcf-4039-8d3e-839cf4b66595&scheme=1)
